# Association of CDX2 and mucin expression with chemotherapeutic benefits in patients with stage II/III gastric cancer

**DOI:** 10.1002/cam4.6379

**Published:** 2023-08-21

**Authors:** Xianchun Gao, Weili Han, Ling Chen, Hongwei Li, Fenli Zhou, Bin Bai, Junya Yan, Yong Guo, Kun Liu, Wenjiao Li, Renlong Li, Qiangqiang Yuan, Jiehao Zhang, Yuanyuan Lu, Xiaodi Zhao, Gang Ji, Mengbin Li, Qingchuan Zhao, Kaichun Wu, Zengshan Li, Yongzhan Nie

**Affiliations:** ^1^ State Key Laboratory of Holistic Integrative Management of Gastrointestinal Cancers and National Clinical Research Center for Digestive Diseases, Xijing Hospital of Digestive Diseases Fourth Military Medical University Xi'an China; ^2^ Department of Health Statistics, Shaanxi Key Laboratory of Free Radical Biology and Medicine and the Ministry of Education Key Lab of Hazard Assessment and Control in Special Operational Environment, School of Preventive Medicine Fourth Military Medical University Xi'an China; ^3^ State Key Laboratory of Holistic Integrative Management of Gastrointestinal Cancers, Department of Pathology, Xijing Hospital and School of Basic Medicine Fourth Military Medical University Xi'an China

**Keywords:** adjuvant chemotherapy, CDX2, gastric cancer, immunohistochemistry, mucin

## Abstract

**Background:**

Better predictors of patients with stage II/III gastric cancer (GC) most likely to benefit from adjuvant chemotherapy are urgently needed. This study aimed to assess the ability of CDX2 and mucin markers to predict prognosis and fluorouracil‐based adjuvant chemotherapy benefits.

**Methods:**

CDX2 and mucin protein expressions were examined by immunohistochemistry and compared with survival and adjuvant chemotherapy benefits in a prospective evaluation cohort of 782 stage II/III GC patients. Then, the main findings were validated in an independent validation cohort (*n* = 386) and an external mRNA sequencing dataset (ACRG cohort, *n* = 193).

**Results:**

In the evaluation cohort, CDX2, CD10, MUC2, MUC5AC, and MUC6 expressions were observed in 59.7%, 26.7%, 27.6%, 55.1%, and 57.7% of patients, respectively. However, only the expression of CDX2 was found to be associated with adjuvant chemotherapy benefits. Most importantly, CDX2‐negative patients had a poorer prognosis when treated with surgery only, while the prognosis of CDX2‐negative and CDX2‐positive patients was similar when receiving postoperative adjuvant chemotherapy. Further analysis revealed that patients with CDX2 negative tumors benefited from chemotherapy (5‐year overall survival rates: 60.0% with chemotherapy vs. 23.2% with surgery‐only, *p* < 0.001), whereas patients with CDX2 positive tumors did not (*p*_interaction_ = 0.004). Consistent results were obtained in the validation and ACRG cohorts.

**Conclusions:**

Negative expression of CDX2 is an independent risk factor for survival in stage II/III GC, but subsequent adjuvant chemotherapy is able to compensate for this unfavorable effect. Therefore, active chemotherapy is more urgent for patients with negative CDX2 expression than for patients with positive CDX2 expression.

## Graphical abstract

CDX2 and mucin protein expressions were examined and compared with survival and adjuvant chemotherapy benefits in a prospective evaluation cohort of 782 stage II/III gastric adenocarcinoma patients. Only the expression of CDX2 was found to be associated with adjuvant chemotherapy benefits. CDX2 negative tumors benefited from chemotherapy, whereas patients with CDX2 positive tumors did not.

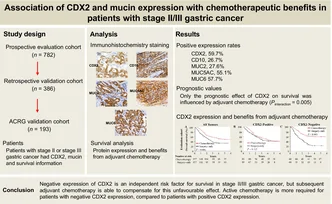

## 1 INTRODUCTION

Gastric cancer (GC) is a leading cause of cancer‐related death worldwide, especially in East Asian regions, including Japan, Korea, and China (18.7 per 100,000 population).^1^ During the past decade, the survival of patients with stage II‐III resectable GC has increased owing to the introduction of adjuvant chemotherapy.^2^ However, this therapeutic advance has been far from enabling patients to obtain significant survival benefits. The benefits of chemotherapy may be offset by its drawbacks. In the follow‐up analysis of the CLASSIC trial, only a moderate absolute survival benefit of 9% (78% vs. 69%) was observed from adjuvant chemotherapy.^2^ Simple and robust predictive biomarkers for the identification of patients with advanced resectable GC who are at high risk for relapse and are likely to benefit from adjuvant or salvage therapy are lacking.

It has been demonstrated that gene expression signatures hold great promise for future research as prognostic classifiers are those derived from stem cells and progenitor cells.^3^ Caudal‐type homeobox transcription factor 2 (CDX2), which mainly regulates intestinal functions in stem/progenitor cells,^4^ is an undoubtedly promising candidate for further analysis and validation. Several studies have demonstrated that loss of CDX2 expression is correlated with poor outcomes in patients with colorectal cancer.^4^, ^5^, ^6^, ^7^ In 2016, Dalerba et al. published a study in *The New England Journal of Medicine* that showed that in addition to being associated with poor survival, a lack of CDX2 expression signature in colon cancer patients has been correlated with improved chemotherapy response.^4^ Subsequent studies validated the prognostic impact of CDX2 in colorectal cancer.^5^, ^6^ Gastric and colorectal cancers share similar biological features. However, the prognostic relevance of CDX2 in GC has not been sufficiently validated and remains controversial.^8^, ^9^ Furthermore, no previous studies have explored the effects of CDX2 on the adjuvant chemotherapy response. Taken together, these results prompted us to further investigate the prognostic value of CDX2 and its impact on the benefits of adjuvant chemotherapy in GC.

Mucins are a family of large glycoproteins that are overexpressed in various aggressive cancers.^10^ It has been increasingly recognized that mucins are associated with cancer stem cell maintenance, disease progression, and chemoresistance.^11^, ^12^ As studies on mucins have developed, the gastric and intestinal phenotypes of gastric adenocarcinoma have been clearly revealed by the expression of different epithelial differentiation markers (MUC5AC and MUC6 as markers for the gastric phenotype, and MUC2 and CD10 as markers for the intestinal phenotype).^13^, ^14^ Each tumor was phenotypically classified into four categories based on immunohistochemistry (IHC) status: intestinal (positive for only intestinal markers), gastric (positive for only gastric markers), gastrointestinal (positive for both gastric and intestinal markers), and unclassified phenotype (negative for all four markers). Although some previous studies have implicated CDX2 and mucin expression in GC,^15^, ^16^ most of them only analyzed the correlation between CDX2 and mucin expression and did not evaluate their prognostic values. In addition, the effects of mucin expression on postoperative chemotherapy response in GC patients are rarely investigated. Thus, there is a need to explore the correlation between specific mucin protein expression and prognosis or response to chemotherapy.

In this work, CDX2 and mucin (CD10, MUC2, MUC5AC, and MUC6) protein expressions were studied in a prospective evaluation cohort of patients with stage II/III gastric adenocarcinoma, and compared with survival, clinicopathologic variables, and benefits from fluorouracil‐based adjuvant chemotherapy in these patients. Then, we validated the main findings in a retrospective validation cohort of randomly selected patients. Simultaneously, data from the Asian Cancer Research Group (ACRG) were analyzed for external validation. Finally, we applied mRNA sequencing data of the ACRG cohort to analyze the mechanism underlying the effects.

## 2 MATERIALS AND METHODS

### 2.1 Data sources

**FIGURE 1:**
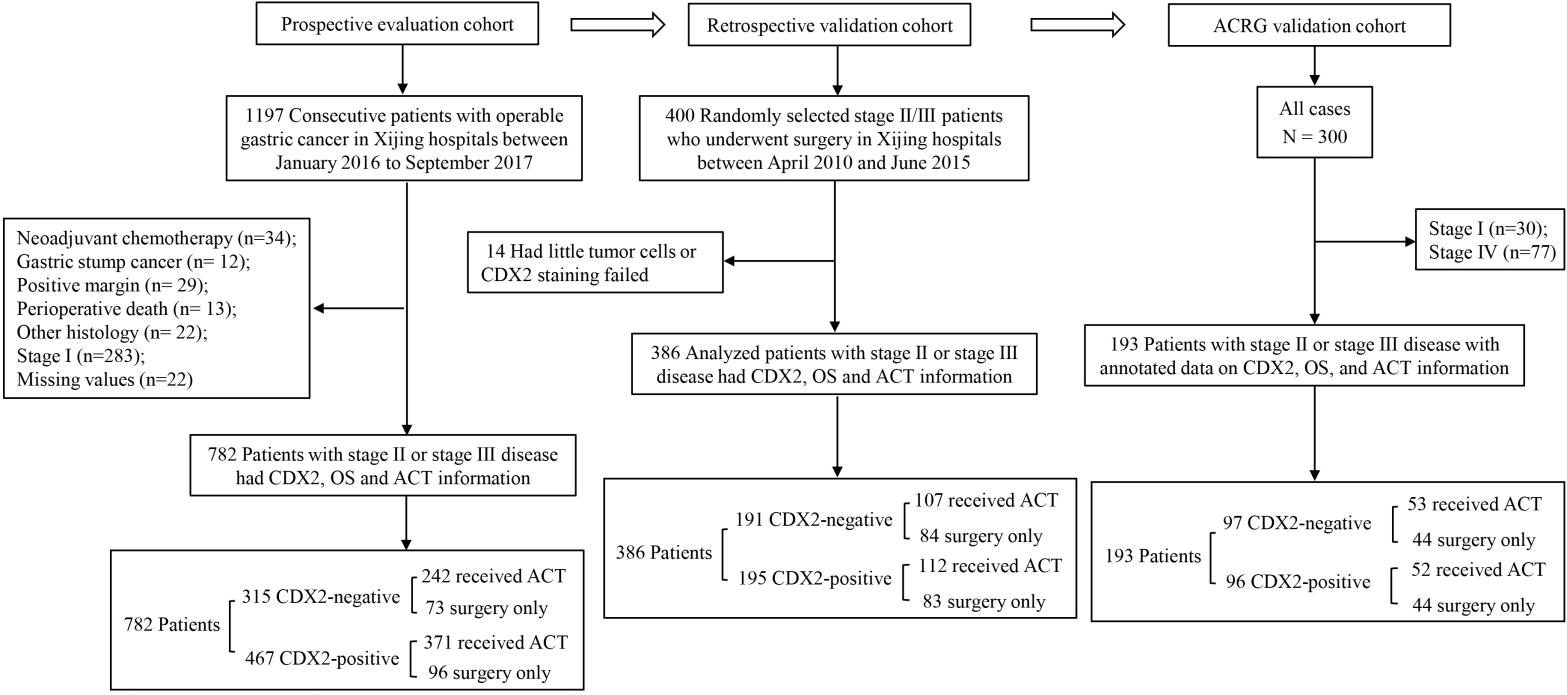
Study flow diagram. ACT, adjuvant chemotherapy.

Three patient cohorts were included in this study: two internal cohorts based on the date of surgery (a prospective evaluation cohort and a retrospective validation cohort) and an external ACRG cohort. The inclusion criteria were as follows: (1) age ≥ 18 years, (2) curative surgical resection with histologically negative resection margins, (3) pathologically confirmed gastric adenocarcinoma, (4) pathologically proven tumor‐node‐metastasis (pTNM) stage II/III GC, and (5) complete follow‐up data. The exclusion criteria were as follows: (1) neoadjuvant therapy before surgery, (2) history of gastrectomy, (3) palliative surgical resection, (4) perioperative death (1 month after surgery), (5) gastric tumors other than adenocarcinoma, (6) pathologically proven TNM stage I GC, and (7) missing values. The flow diagram is presented in Figure 1.

Finally, in the prospective evaluation cohort, a preliminary test involving 782 consecutive patients who underwent surgery between January 2016 and September 2017 in Xijing Hospital was conducted. Subsequently, we validated the main findings in a retrospective validation cohort of 386 randomly selected patients who underwent surgery between April 2010 and June 2015 (follow‐up over 5 years) in Xijing Hospital and an external public ACRG cohort (*n* = 193). A total of 1361 patients with gastric adenocarcinoma were enrolled in this study (Figure 1). The last follow‐up date for internal patients was March 7, 2022. This study was approved by the Ethical Committee of Xijing Hospital.

The clinical information of the internal populations was drawn from the Xijing Hospital of Digestive Diseases Gastric Cancer Database (XJHDDGCD).^17^, ^18^ This database contains prospectively collected clinicopathologic data, biological specimens, and follow‐up information on patients who were admitted to Xijing Hospital. Tissue samples from gastric cancer patients eligible for inclusion can be randomly selected for this study. This database was founded in 2008 and is now one of the largest GC databases in China. By December 2021, the database included more than 12,000 patients who received a diagnosis of stage I to IV GC. All patients provided written informed consent before surgery for the use of their clinicopathological data and resected samples. Most of the patients underwent surgical resection and had detailed clinicopathological and follow‐up information. Patients were followed prospectively every 6‐ to 12‐month interval by telephone, and the results of laboratory tests, imaging examinations, and gastroscopy were recorded. The clinical information and mRNA sequencing data of the ACRG cohort and The Cancer Genome Atlas (TCGA) cohort were downloaded from http://www.ncbi.nlm.nih.gov/geo/ on March 1, 2021. Consistently, only patients with II‐III disease were included. Because the pTNM staging system changed during the study period, it was uniformly adjusted according to the 8th edition of the AJCC staging manual for patients in the evaluation cohort and validation cohort. However, due to the lack of original data on the T stage and N stage of patients in the ACRG cohort, the TNM staging of patients in the ACRG cohort was based on the 6th TNM staging.

### 2.2 Adjuvant chemotherapy treatment

Fluorouracil‐based adjuvant chemotherapy was routinely recommended for patients with stage II‐III GC in accordance with Chinese Gastric Cancer guidelines. In this study, the final decision about adjuvant chemotherapy was made after additional investigation of the patients' actual clinical condition and discussion with the patient. No restriction was placed regarding the interval between surgery and initiation of adjuvant therapy. In the evaluation cohort, 613 patients received adjuvant chemotherapy, of whom 441 (71.9%) received at least 6 cycles of chemotherapy; in terms of adjuvant chemotherapy regimens, 364 (59.4%) received multiagent chemotherapy, 190 (31.0%) received single‐agent chemotherapy and 59 patients (9.6%) did not have details of agents. In the validation cohort, 219 patients received adjuvant chemotherapy, of which 142 (64.8%) received at least 6 cycles of chemotherapy; in terms of adjuvant chemotherapy regimens, 139 (63.5%) received multiagent chemotherapy, 54 (24.7%) received single‐agent chemotherapy and 26 patients (11.9%) did not have details of agents. In the ACRG cohort, 105 patients received adjuvant chemotherapy, of which 69 (65.7%) received multiagent chemotherapy, and 36 (34.3%) received single‐agent chemotherapy; the chemotherapy cycles were not available. In the TCGA cohort, adjuvant chemotherapy information was not available.

### 2.3 Immunohistochemistry staining

For each sample, 3‐μm serial sections were excised from selected formalin‐fixed, paraffin‐embedded tumor blocks for IHC analyses. For CDX2 staining, a CDX2 ready‐to‐use antibody (clone AMT28, monoclonal mouse, Maixin, Fuzhou, China) was applied. The mucin phenotype was examined using a CD10 ready‐to‐use antibody (clone MX002, monoclonal mouse, Maixin, Fuzhou, China), a MUC2 ready‐to‐use antibody (clone M53, monoclonal mouse, Maixin, Fuzhou, China), a MUC5AC ready‐to‐use antibody (clone 45 M1, monoclonal mouse, Maixin, Fuzhou, China), and a MUC6 ready‐to‐use antibody (clone MRQ‐20, monoclonal mouse, Maixin, Fuzhou, China). Tissue slides were stained on a Bond‐Max automatic staining system according to the manufacturer's instructions (Leica), and antigen detection was visualized by the Bond Polymer Refine Detection Kit (Leica).

For the evaluation cohort, CDX2 and mucin staining were performed prospectively within 1 week after surgery. For the validation cohort, only CDX2 staining was performed retrospectively.

### 2.4 Evaluation of IHC staining results

In the prospective evaluation cohort, all tissue sections were scored for CDX2 and mucin IHC expression by an experienced gastrointestinal cancer pathologist who was blinded to the clinicopathologic information (CL, LZS). To test the reproducibility of the CDX2 score, in the retrospective validation cohort, all tissue sections were scored for CDX2 expression by two independent investigators (ZFL and LHW). The association between CDX2 and mucin status and clinical outcome was evaluated by an investigator who did not take part in the scoring process.

**FIGURE 2:**
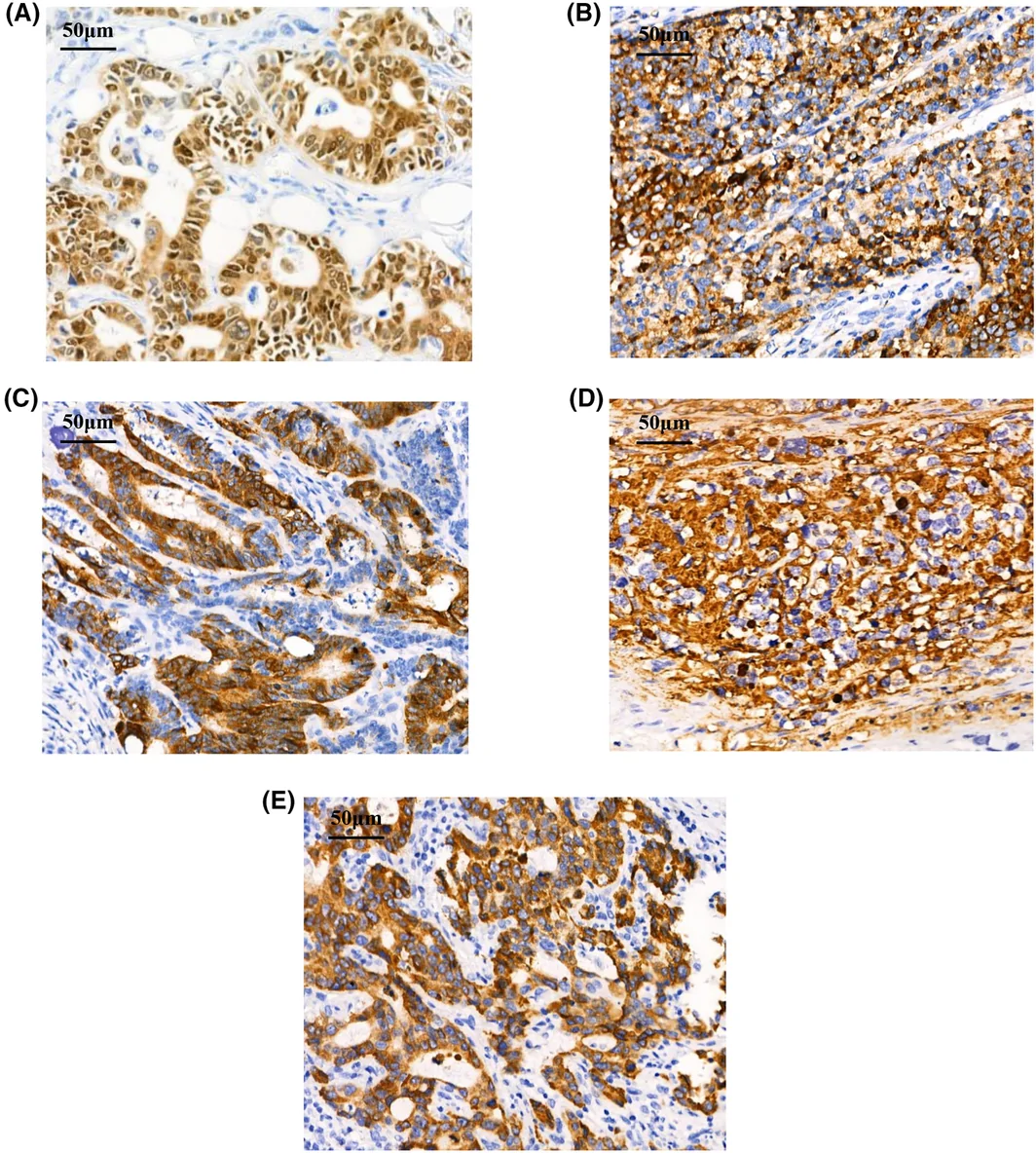
The degree of CDX2 and mucin expression in gastric adenocarcinoma tissue sections detected by immunohistochemistry. (A) (CDX2), (B) (CD10), (C) (MUC2), (D) (MUC5AC), and (E) (MUC6) (magnification ×200).

Only nuclear CDX2 staining was considered positive, and only tumor cells were scored in the whole tissue section (intestinal metaplasia was excluded). The intensity of staining was graded on a scale of 0 to 2. We defined the score within the whole tissue section according to Dalerba et al.^4^ as follows: 0 = no reactivity of tumor cells; 1+ = faint/barely perceptible nuclear reactivity in a minority of tumor cells; 2+ = moderate or strong nuclear reactivity in a majority of tumor cells. Representative images of the corresponding IHC staining scores for CDX2 expression are shown in Figure 2. For the final scores, an IHC score of 0 or 1+ was defined as negative (loss) and an IHC score of 2+ was defined as positive.

The expression of CD10 (an intestinal brush border marker), MUC2 (an intestinal goblet cell marker), MUC5AC (a gastric foveolar epithelium marker), or MUC6 (a marker of gastric mucous neck cells and pyloric glands) was regarded as positive when ≥10% of tumor cells were stained positive (Figure 2).^19^

### 2.5 Molecular characteristics and tumor microenvironment

In the signaling pathway analysis, differential gene expression analysis was performed on all genes to analyze the samples with high (*n* = 96) and low (*n* = 97) CDX2 expression. For functional profiling of changes in mRNA, gene set enrichment analysis (GSEA) was performed to investigate the underlying biological pathways. CDX2 expression levels were correlated with multidrug resistance‐associated genes transcript levels by Spearman's rank correlation coefficients. To estimate the tumor microenvironment, we used an algorithm called ESTIMATE to assess the stromal and immune cells in gastric tumor tissues based on the mRNA sequencing data of the ACRG cohort. To validate the findings, IHC was performed to detect the expression of CD8 in representative CDX2 positive (*n* = 30) and negative tumor tissues (*n* = 30) from patients with GC (ab17147, Abcam).

### 2.6 Follow‐up and statistical analysis

Overall survival (OS) was calculated from the date of primary surgery to patient death from any cause that was scored as an event. Recurrence‐free survival (RFS) was defined as the time from the date of primary surgery until the first evidence of relapse that was scored as an event. Patients without any event were censored at the last follow‐up date. Due to the lack of available disease recurrence/distant metastasis information in the ACRG cohort, we could only assess the association between CDX2 expression and OS.

The kappa value was calculated to assess the interobserver agreement on the expression of CDX2. The relationships between CDX2 and mucin status and clinicopathological factors were analyzed using Pearson's chi‐square test. The probability of survival for the different patient subgroups was assessed using the Kaplan–Meier method and log‐rank tests. Adjusted multivariate Cox proportional hazards regression models were used to evaluate independent prognostic values. The interaction between CDX2 and mucin status and treatment was also analyzed using the Cox model. To minimize the influence of the difference in baseline characteristics on our results, we used propensity score matching to compare OS between the adjuvant chemotherapy and surgery‐only groups. In this model, we explored clinically relevant variables to estimate the propensity scores.

The statistical analyses were conducted using SPSS (version 24, SPSS Inc), R software (version 3.3.2, statistical software), GraphPad Prism (version 8.0.2 GraphPad Software), and GSEA (version 4.0.2 Broad Institute Inc). A two‐sided *p* value <0.05 was considered significant.

## 3 RESULTS

### 3.1 Patients

The baseline clinicopathological characteristics of the evaluation, validation, and ACRG cohorts are summarized in Table 1. In the evaluation cohort, of 782 patients, 351 deaths occurred, with a median follow‐up of 4.11 (95% confidence interval [CI] 4.10–4.12) years. In the validation cohort, of 386 patients, 211 deaths occurred, with a median follow‐up of 6.00 (95% CI 5.94–6.05) years. In the ACRG cohort, of 193 patients, 84 deaths occurred, in a median follow‐up of 7.05 (95% CI 6.74–7.36) years.

**TABLE 1:** The distributions of clinicopathologic characteristics of gastric cancer patients in the three cohorts.

Parameter	Evaluation cohort n = 782 (%)	Validation cohort n = 386 (%)	ACRG cohort n = 193 (%)
Age (years)			
≥65	213 (27.2)	136 (35.2)	89 (46.1)
<65	569 (72.8)	250 (64.8)	104 (53.9)
Sex			
Male	597 (76.3)	297 (76.9)	138 (71.5)
Female	185 (23.7)	89 (23.1)	55 (28.5)
Primary tumor location			
Gastric	520 (66.5)	251 (65.0)	170 (88.1)
GE junction	262 (33.5)	135 (35.0)	22 (11.4)
Missing value	—	—	1 (0.5)
pT stage*			
T1 or T2	85 (10.9)	19 (4.9)	134 (69.4)
T3 or T4	697 (89.1)	367 (95.1)	59 (30.6)
pN stage*			
N0 or N1	295 (37.7)	146 (37.8)	126 (65.3)
N2 or N3	487 (62.3)	240 (62.2)	67 (34.7)
Histopathological differentiation			
High/middle	138 (17.6)	128 (33.2)	—
Poor	644 (82.4)	258 (66.8)	—
Lauren's classification			
Diffuse	—	—	99 (51.3)
Intestinal	—	—	88 (45.6)
Mixed	—	—	6 (3.1)
Perineural invasion			
No	48 (6.1)	45 (11.7)	—
Yes	734 (93.9)	341 (88.3)	—
Lymphovascular invasion			
No	171 (21.9)	124 (32.1)	—
Yes	611 (78.1)	262 (67.9)	—
Examined lymph nodes			
≥20	630 (80.6)	342 (88.6)	—
<20	152 (19.4)	44 (11.4)	—
CEA, ng/mL			
<5	623 (79.7)	278 (72.0)	
≥5	159 (20.3)	108 (28.0)	
CA19‐9, U/mL			
<27	599 (76.6)	270 (69.9)	
≥27	183 (23.4)	116 (30.1)	
Pathologic stage*			
Stage II	257 (32.9)	109 (28.2)	97 (50.3)
Stage III	525 (67.1)	277 (71.8)	96 (49.7)
Treatment arm			
Surgery‐only	169 (21.6)	167 (43.3)	88 (45.6)
Surgery + chemotherapy	613 (78.4)	219 (56.7)	105 (54.4)

* AJCC 8th for the evaluation cohort and validation cohort, AJCC 6th for the ACRG cohort.

Abbreviations: GE junction, gastroesophageal junction; pN stage, pathological nodal stage; pT stage, pathological tumor stage.

### 3.2 Expression levels of CDX2 and mucin

In the evaluation cohort, CDX2, CD10, MUC2, MUC5AC, and MUC6 positive expression levels were observed in 59.7% (467 patients), 26.7% (209), 27.6% (216), 55.1% (431), and 57.7% (451) of patients, respectively. CDX2 expression in gastric adenocarcinoma was found to be associated with well‐differentiated tumors (*p* = 0.027). Patients with positive CD10 expression tended to receive adjuvant chemotherapy (*p* = 0.046). Patients with positive MUC2 expression tended to demonstrate lymphovascular invasion (*p* = 0.010) and develop tumors with a high TNM classification (*p* = 0.002). MUC5AC‐positive adenocarcinoma frequently developed in the gastric location (*p* = 0.041) and tended to demonstrate poorly differentiated (*p* = 0.023) and lymphovascular invasion tumors (*p* = 0.013). Patients with positive MUC6 expression tended to demonstrate perineural invasion (*p* = 0.004) and lymphovascular invasion (*p* = 0.017) (Table 2).

**TABLE 2:** Clinical and demographic variables by CDX2 and mucin expression.

	No. of positive patients					
Parameter	CDX2^a^	CD10^a^	MUC2^a^	MUC5AC^a^	MUC6^a^	CDX2^b^
	*n* = 467 (%)	n = 209 (%)	n = 216 (%)	n = 431 (%)	n = 451 (%)	n = 195 (%)
Age (years)						
≥65	126 (59.2)	55 (25.8)	67 (31.5)	119 (55.9)	132 (62.0)	72 (52.9)
<65	341 (59.9)	154 (27.1)	149 (26.2)	312 (54.8)	319 (56.1)	123 (49.2)
*p* value	0.844	0.726	0.142	0.795	0.137	0.483
Sex						
Male	364 (61.0)	160 (26.8)	174 (29.1)	324 (54.3)	336 (56.3)	123 (49.2)
Female	103 (55.7)	49 (26.5)	42 (22.7)	107 (57.8)	115 (62.2)	72 (52.9)
*p* value	0.199	0.933	0.087	0.394	0.157	0.338
Primary tumor location						
Gastric	318 (61.2)	133 (25.6)	145 (27.9)	300 (57.7)	304 (58.5)	124 (49.4)
GE junction	149 (56.9)	76 (29.0)	71 (27.1)	131 (50.0)	147 (56.1)	71 (52.6)
*p* value	0.249	0.306	0.817	0.041	0.529	0.550
pT stage (AJCC 8th)						
T1 or T2	50 (58.8)	26 (30.6)	25 (29.4)	54 (63.5)	54 (63.5)	12 (63.2)
T3 or T4	417 (59.8)	183 (26.3)	191 (27.4)	377 (54.1)	397 (57.0)	183 (49.9)
*p* value	0.859	0.394	0.696	0.099	0.247	0.258
pN stage (AJCC 8th)						
N0 or N1	176 (59.7)	87 (29.5)	73 (24.7)	156 (52.9)	159 (53.9)	76 (52.1)
N2 or N3	291 (59.8)	122 (25.1)	143 (29.4)	275 (56.5)	292 (60.0)	119 (49.6)
*p* value	0.980	0.174	0.162	0.328	0.096	0.638
Histopathological differentiation						
High/middle	94 (68.1)	42 (30.4)	29 (21.0)	64 (46.4)	78 (56.5)	74 (57.8)
Poor	373 (57.9)	167 (25.9)	187 (29.0)	367 (57.0)	373 (57.9)	121 (46.9)
*p* value	0.027	0.278	0.056	0.023	0.763	0.043
Perineural invasion						
No	27 (56.3)	14 (29.2)	10 (20.8)	23 (47.9)	18 (37.5)	22 (48.9)
Yes	440 (59.9)	195 (26.6)	206 (28.1)	408 (55.6)	433 (59.0)	173 (50.7)
*p* value	0.613	0.693	0.278	0.301	0.004	0.816
Lymphovascular invasion						
No	104 (60.8)	51 (29.8)	34 (19.9)	80 (46.8)	85 (49.7)	71 (57.3)
Yes	363 (59.4)	158 (25.9)	182 (29.8)	351 (57.4)	366 (59.9)	124 (47.3)
*p* value	0.740	0.300	0.010	0.013	0.017	0.068
Examined lymph nodes						
≥20	380 (60.3)	164 (26.0)	179 (28.4)	345 (54.8)	354 (56.2)	173 (50.7)
<20	87 (57.2)	45 (29.6)	37 (24.3)	86 (56.6)	97 (63.8)	22 (48.9)
*p* value	0.487	0.372	0.314	0.686	0.088	0.816
CEA, ng/mL						
<5	366 (58.7)	162 (26.0)	157 (25.2)	338 (54.3)	367 (58.9)	143 (51.4)
≥5	101 (63.5)	47 (29.6)	59 (37.1)	93 (58.5)	84 (52.8)	52 (48.1)
*p* value	0.273	0.366	0.003	0.338	0.166	0.562
CA19‐9, U/mL						
<27	353 (58.9)	166 (27.7)	148 (24.7)	320 (53.4)	352 (58.8)	145 (53.7)
≥27	114 (62.3)	43 (23.5)	68 (37.2)	111 (60.7)	99 (54.1)	50 (43.1)
*p* value	0.417	0.259	0.001	0.085	0.263	0.056
Pathologic stage (AJCC 8th)						
Stage II	145 (56.4)	74 (28.8)	53 (20.6)	137 (53.3)	139 (54.1)	60 (55.0)
Stage III	322 (61.3)	135 (25.7)	163 (31.0)	294 (56.0)	312 (59.4)	135 (48.7)
*p* value	0.188	0.361	0.002	0.477	0.155	0.264
Treatment arm						
Surgery‐only	96 (56.8)	35 (20.7)	44 (26.0)	89 (52.7)	98 (58.0)	83 (49.7)
Surgery + chemotherapy	371 (60.5)	174 (28.4)	172 (28.1)	342 (55.8)	353 (57.6)	112 (51.1)
*p* value	0.383	0.046	0.602	0.469	0.925	0.779

Abbreviations: GE junction, gastroesophageal junction; pN stage, pathological nodal stage; and pT stage, pathological tumor stage.

^a^ Evaluation cohort.

^b^ Validation cohort.

We next investigated the association between CDX2 status and four epithelial differentiation markers for phenotypic classification. Of the four markers, CDX2 status was significantly correlated with CD10 and MUC2 expression (*p* < 0.001). The rate of CDX2 positive expression in the tumor sections with positive CD10 and MUC2 expression was significantly higher than that in sections with negative CD10 and MUC2 expression. CDX2 status was correlated with MUC5AC expression, although the correlation was not statistically significant (*p* = 0.050). No correlation was found between MUC6 and CDX2 expression. When the mucin phenotype was classified into four subtypes (intestinal, gastric, gastrointestinal, and unclassified phenotypes), patients exhibiting the intestinal and gastrointestinal phenotypes showed high CDX2‐positive ratios, and the difference among the phenotypes was significant (*p* < 0.001) (Figure S1).

In the validation cohort, CDX2‐positive expression was observed in 50.5% (195) of patients. Furthermore, two investigators scored CDX2 expression independently based on the same criteria in 386 gastric adenocarcinoma samples in the validation cohort. The test for interobserver agreement showed good consistency in terms of the final CDX2 status of individual patients (kappa value 0.927) (Table S1). Nearly half of the patients in both the evaluation and validation cohorts were CDX2 positive, so the cut‐off value for the classification of CDX2 high (positive)/low (negative) subgroups was the median value in the ACRG cohort. Therefore, we classified 96 patients (49.7%) in the ACRG cohort as CDX2‐high.

### 3.3 Effects of CDX2 and mucin expression on patient survival

**FIGURE 3:**
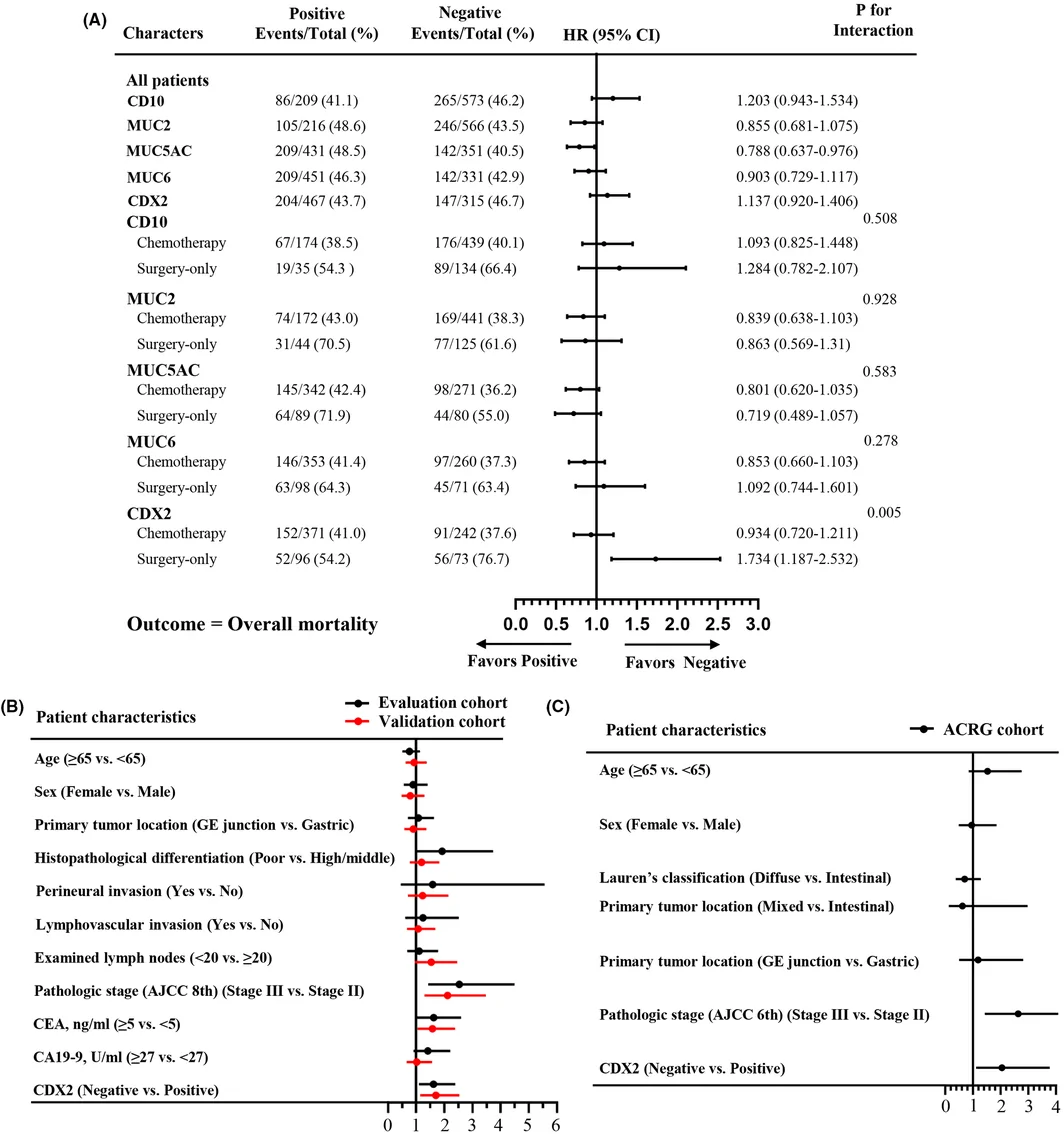
Prognostic analysis of different CDX2 and mucin expression subgroups. (A) Treatment interaction with CDX2 and mucin expression for overall survival. (B and C) Cox regression analysis of prognostic factors for overall survival in surgery‐only patients (B: in the evaluation cohort and validation cohort, C: in the ACRG cohort). CI, confidence intervals; GE junction, gastroesophageal junction; HR, hazard ratio.

In the evaluation cohort, only MUC5AC expression was associated with better OS (hazard ratio [HR] = 0.788, 95% CI, 0.637–0.976, *p* = 0.029), whereas CDX2, CD10, MUC2, and MUC6 expressions were not significantly associated with the prognosis of gastric adenocarcinoma (all *p* > 0.05) (Figure 3A).

**FIGURE 4:**
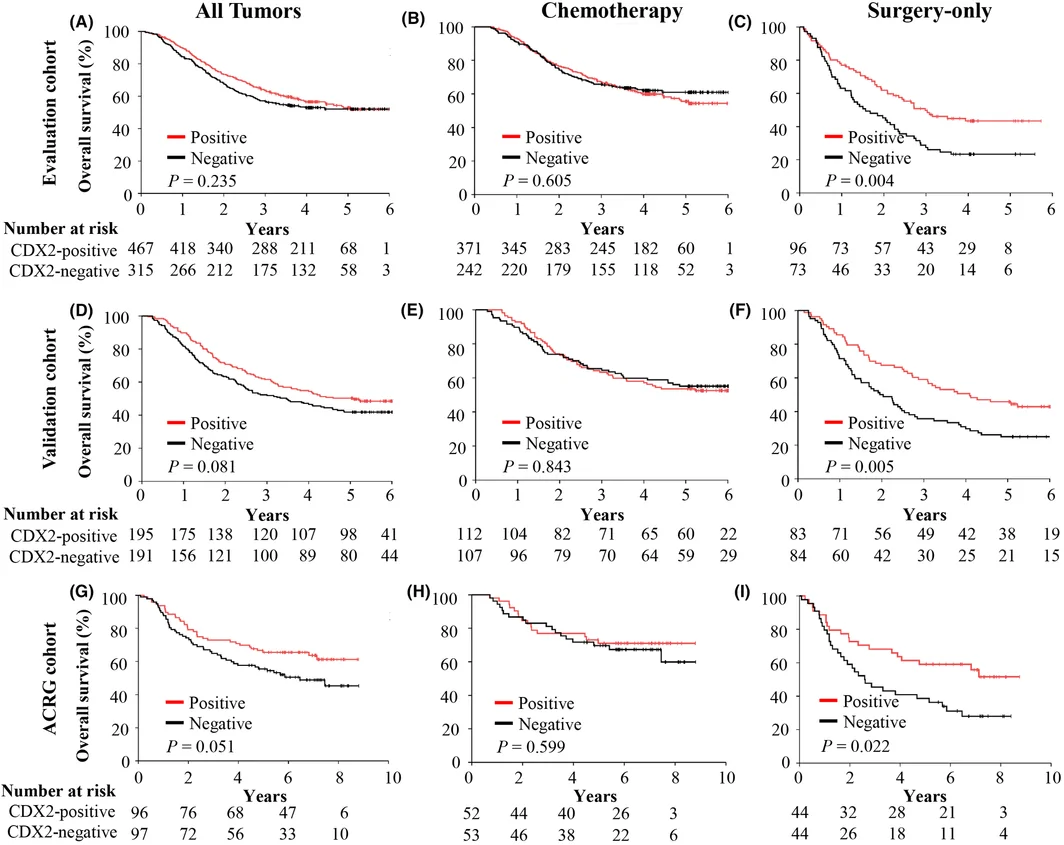
Relationship between CDX2 status and patient overall survival. (A–C) Kaplan–Meier plots for overall survival in the evaluation cohort according to CDX2 status (A: all tumors, B: adjuvant chemotherapy subgroup, C: surgery‐only subgroup). (D–F) Kaplan–Meier plots for overall survival in the validation cohort according to CDX2 status (D: all tumors, E: adjuvant chemotherapy subgroup, F: surgery‐only subgroup). (G–I) Kaplan–Meier plots for overall survival in the ACRG cohort according to CDX2 status (G: all tumors, H: adjuvant chemotherapy subgroup, I: surgery‐only subgroup).

To evaluate whether the effects of CDX2 and mucin expression on survival were confounded by adjuvant chemotherapy, we examined the association between CDX2 and mucin expressions and survival in subgroup analyses based on adjuvant chemotherapy (Figure 3A). In the surgery‐only subgroup, a CDX2‐negative status was associated with a worse OS rate than a CDX2‐positive status (HR = 1.734, 95% CI: 1.187–2.532, *p* = 0.004; Figures 3A and 4C). In contrast, there was no correlation between CDX2 status and outcomes in the adjuvant chemotherapy subgroup (HR = 0.934, 95% CI: 0.720–1.211, *p* = 0.605) (Figures 3A and 4B), indicating that adjuvant chemotherapy could compensate for the unfavorable effect of CDX2‐negative status on survival. A test for the interaction between the biomarker and the treatment revealed that the prognostic effect of CDX2 on OS was influenced by adjuvant chemotherapy (*p* = 0.005 for the interaction) (Figure 3A). In contrast, CD10, MUC2, MUC5AC, and MUC6 expressions were not significantly associated with the prognosis of gastric adenocarcinoma in either the adjuvant chemotherapy subgroup or the surgery‐only subgroup (all *p* > 0.05). Furthermore, a test for the interaction between the biomarker and the treatment revealed that the prognostic effect of mucin (CD10, MUC2, MUC5AC, and MUC6) on OS was not influenced by adjuvant chemotherapy (all *p* > 0.05 for the interaction) (Figure 3A).

To verify the robustness of the results, we validated the association of CDX2 expression with postoperative prognosis in an independent validation cohort at the protein level and an external ACRG cohort at the mRNA level. Consistent results were obtained at both the mRNA and protein levels. The expression of CDX2 in stage II/III patients had no significant correlation with prognosis in the unstratified analysis of each whole cohort (all *p* > 0.05) (Figure 4A,D,G). An analysis stratified by adjuvant chemotherapy showed that CDX2‐negative status was associated with a worse OS rate than CDX2‐positive status in both the validation cohort (HR = 1.729, 95% CI: 1.183–2.526, *p* = 0.005) (Figure 4F) and ACRG cohort (HR = 1.933, 95% CI: 1.099–3.400, *p* = 0.022) (Figure 4I) in the surgery‐only subgroup; in contrast, there was no correlation between CDX2 status and outcomes in the adjuvant chemotherapy subgroup in either cohort (both *p* > 0.05) (Figure 4E,H). In addition, the effect of CDX2 expression on RFS was similar to that on OS in the evaluation and validation cohorts (Figure S2). Subsequently, based on the TCGA cohort, we tried to evaluate the prognostic value of CDX2 in patients with different TCGA classifications. High CDX2 expression was associated with a better prognosis among patients with MSS (microsatellite stability) and EBV (Epstein–Barr virus)‐negative GC, although the difference observed nonetheless failed to reach statistical significance (Figure S3B). In contrast, CDX2 expression did not correlate with prognosis among patients with MSI or EBV‐positive GC (Figure S3C).

To evaluate whether CDX2 status was an independent prognostic factor in the surgery‐only subgroup, we analyzed the prognostic relevance of CDX2 using Cox multivariate proportional hazards models adjusted for the following risk factors: age, sex, histopathological differentiation, primary tumor location, perineural invasion, lymphovascular invasion, number of examined lymph nodes, and pTNM stage (AJCC 8th edition) in the internal evaluation and validation cohorts; adjusted for the following risk factors: age, sex, Lauren's classification, primary tumor location, and pTNM stage (AJCC 6th edition) in the ACRG cohort. Although cancer stage was a powerful prognostic factor, CDX2 status was also an independent prognostic factor (Figure 3B,C). The CDX2 status HRs for OS in the evaluation cohort (HR = 1.638, 95% CI, 1.114–2.407, *p* = 0.012) were similar to those in the validation cohort (HR = 1.647, 95% CI, 1.111–2.441, *p* = 0.013) and the ACRG cohort (HR = 2.053, 95% CI, 1.115–3.779, *p* = 0.021).

### 3.4 CDX2 expression and benefits from adjuvant chemotherapy

To confirm the relevance of CDX2 expression in the benefits from adjuvant chemotherapy, we investigated the association between CDX2 status and outcomes among patients who did or did not receive adjuvant chemotherapy. To mitigate the effects of differences in baseline characteristics among patients with and without adjuvant chemotherapy, we adjusted for confounding factors using propensity score matching. After matching, the baseline characteristics of the two groups of patients were similar (Table S2–S4).

**FIGURE 5:**
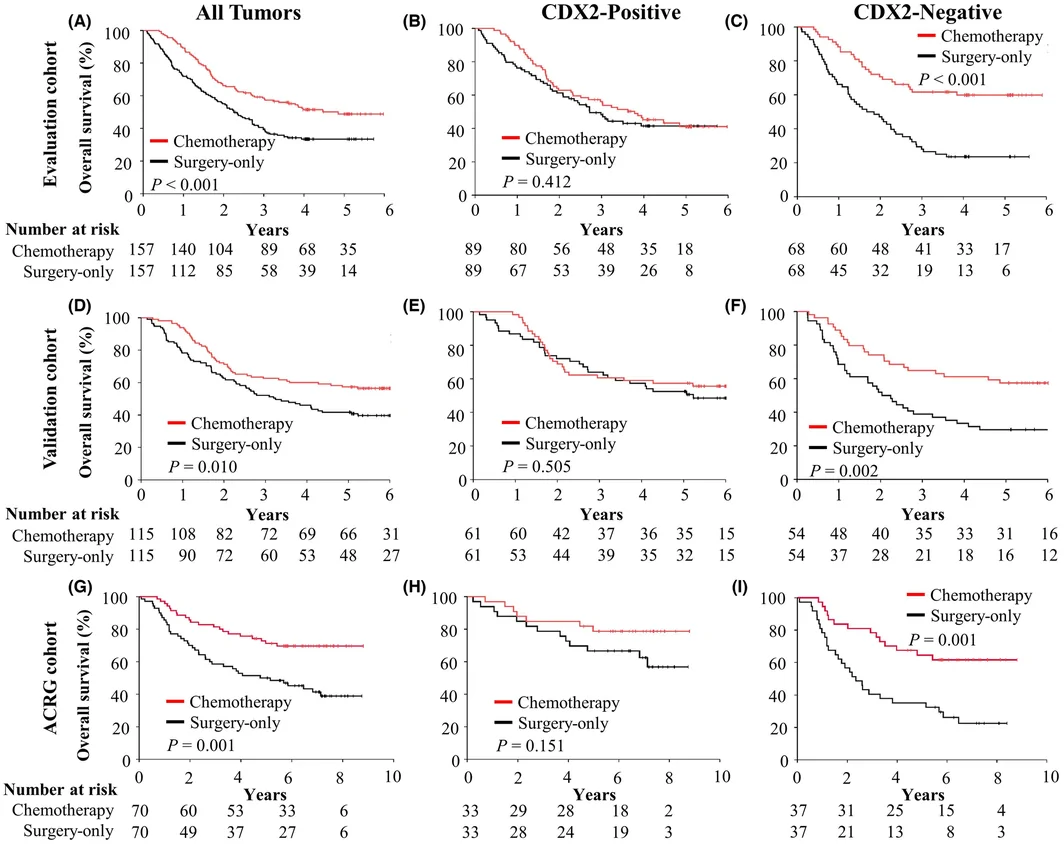
Association between CDX2 status and benefit from adjuvant chemotherapy after propensity score matching. (A–C) Kaplan–Meier plots for overall survival according to treatment in the evaluation cohort (A: all tumors, B: CDX2‐positive subgroup, C: CDX2‐negative subgroup). (D–F) Kaplan–Meier plots for overall survival according to treatment in the validation cohort (D: all tumors, E: CDX2‐positive subgroup, F: CDX2‐negative subgroup). (G–I) Kaplan–Meier plots for overall survival according to treatment in the ACRG cohort (G: all tumors, H: CDX2‐positive subgroup, I: CDX2‐negative subgroup).

The results from the subset analysis using CDX2 status revealed that adjuvant chemotherapy was significantly associated with a higher OS rate in the evaluation (5‐year OS rates: 60.0% with chemotherapy vs. 23.2% with surgery‐only, *p* < 0.001), validation (5‐year OS rates: 57.4% with chemotherapy vs. 29.6% with surgery‐only, *p* = 0.002) and ACRG (5‐year OS rates: 64.8% with chemotherapy vs. 35.1% with surgery‐only, *p* = 0.001) cohorts among those with CDX2‐negative tumors (Figure 5C,F,I). However, for patients with CDX2‐positive tumors, adjuvant chemotherapy was not associated with any improvement in OS in all three cohorts (all *p* > 0.05) (Figure 5B,E,H). A test for the interaction between CDX2 status and treatment revealed that CDX2‐negative patients benefited more from treatment with adjuvant chemotherapy than CDX2‐positive patients in the evaluation (*p* = 0.004 for the interaction). However, due to the relatively small sample size, there may have been insufficient power to detect the interaction between CDX2 status and chemotherapy in the validation and ACRG cohorts (both *p* > 0.05 for the interaction). We obtained similar results for RFS (Figure S4). Consequently, these results suggest that patients with CDX2‐positive tumors are unlikely to benefit from the addition of adjuvant chemotherapy to radical operation, whereas patients with CDX2‐negative tumors derive considerably more benefit.

### 3.5 Molecular characteristics of different CDX2 expression subgroups

**FIGURE 6:**
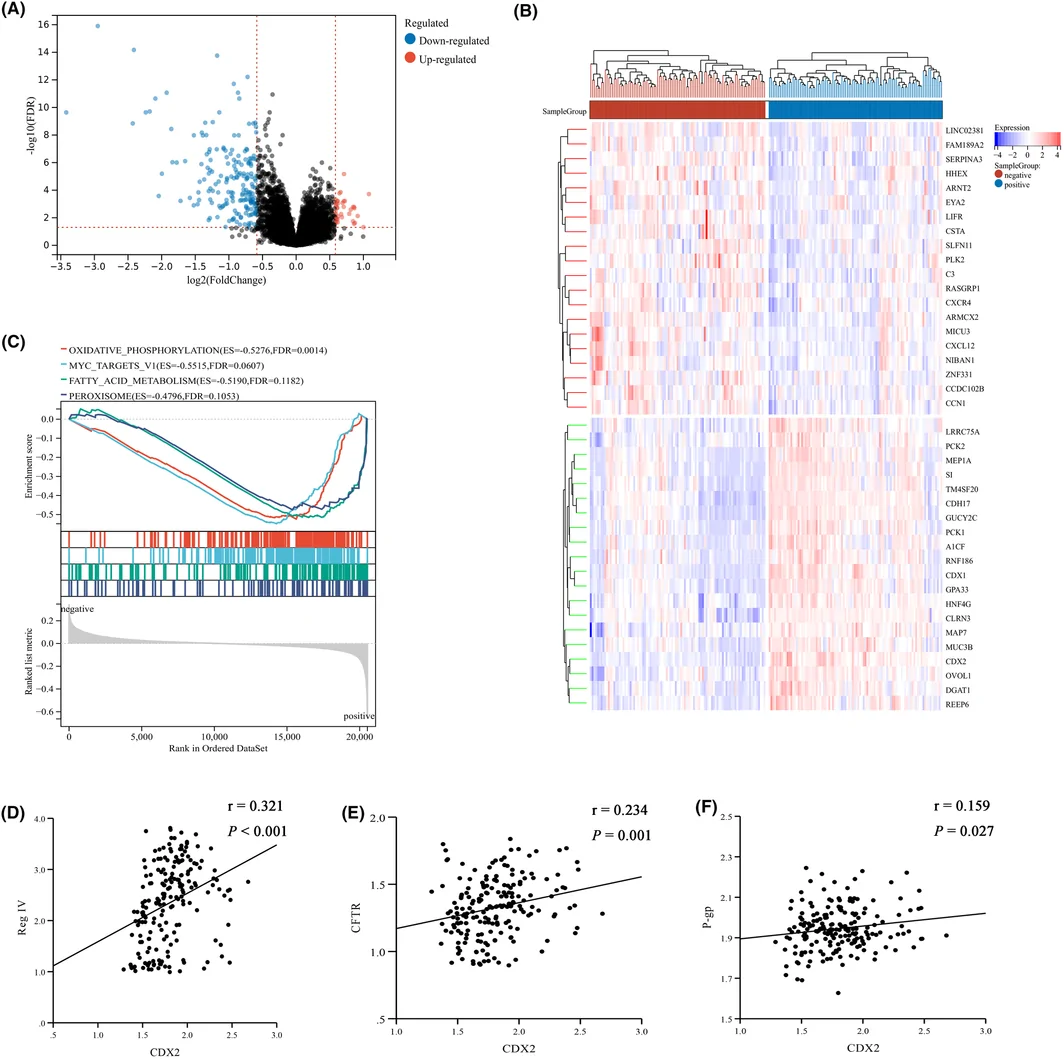
Molecular characteristics of different CDX2 subgroups. (A)Volcano plot of differentially expressed mRNAs in ACRG cohort. Red and blue dots show upregulated and downregulated genes, respectively. (B) Heat map showing expression fold changes of the top 20 most significant up and downregulated genes. Genes upregulated are shown in red and downregulated in blue, as fold changes. (C) Gene sets enriched in the CDX2‐positive subgroup (*p* < 0.05, FDR < 0.25). (D–F) The relationship between CDX2 and its regulatable multidrug resistance‐associated genes expression analyzed by Spearman's rank correlation coefficients.

Based on the RNA‐sequencing data of patients with stage II/III gastric cancer from the public ACRG cohort, there were 39 genes upregulated and 194 genes downregulated in the CDX2‐negative subgroup when compared with the CDX2‐positive subgroup (*p* < 0.05, false discovery rate <0.25) (Figure 6A). Expression fold changes of the top 20 most significant up and downregulated genes are shown in the plot heatmap (Figure 6B). Then GSEA was performed to determine the gene sets enriched in different CDX2 expression subgroups. The gene sets of the CDX2‐positive samples were mainly enriched in oxidative phosphorylation, MYC targets, fatty acid metabolism, and peroxisome‐related pathways (Figure 6C), while no pathway gene sets were positively enriched with statistical significance in the gene sets of the CDX2‐negative samples.

Next, we explore the relationship between CDX2 and its regulatable multidrug resistance‐associated genes expression. As a result, the CDX2 expression was significantly correlated with regenerating gene 4 (Reg IV) (*r* = 0.321, *p* < 0.001), cystic fibrosis transmembrane conductance regulator (CFTR) (*r* = 0.234, *p* = 0.001) and P‐glycoprotein (P‐gp) (*r* = 0.159, *p* = 0.027), shown in Figure 6D–F. Therefore, we suggested that the negative effect of high CDX2 expression on fluorouracil‐based adjuvant chemotherapy might result from its positive regulation on these multidrug resistance‐associated genes.

### 3.6 Immune characteristics of different CDX2 expression groups

**FIGURE 7:**
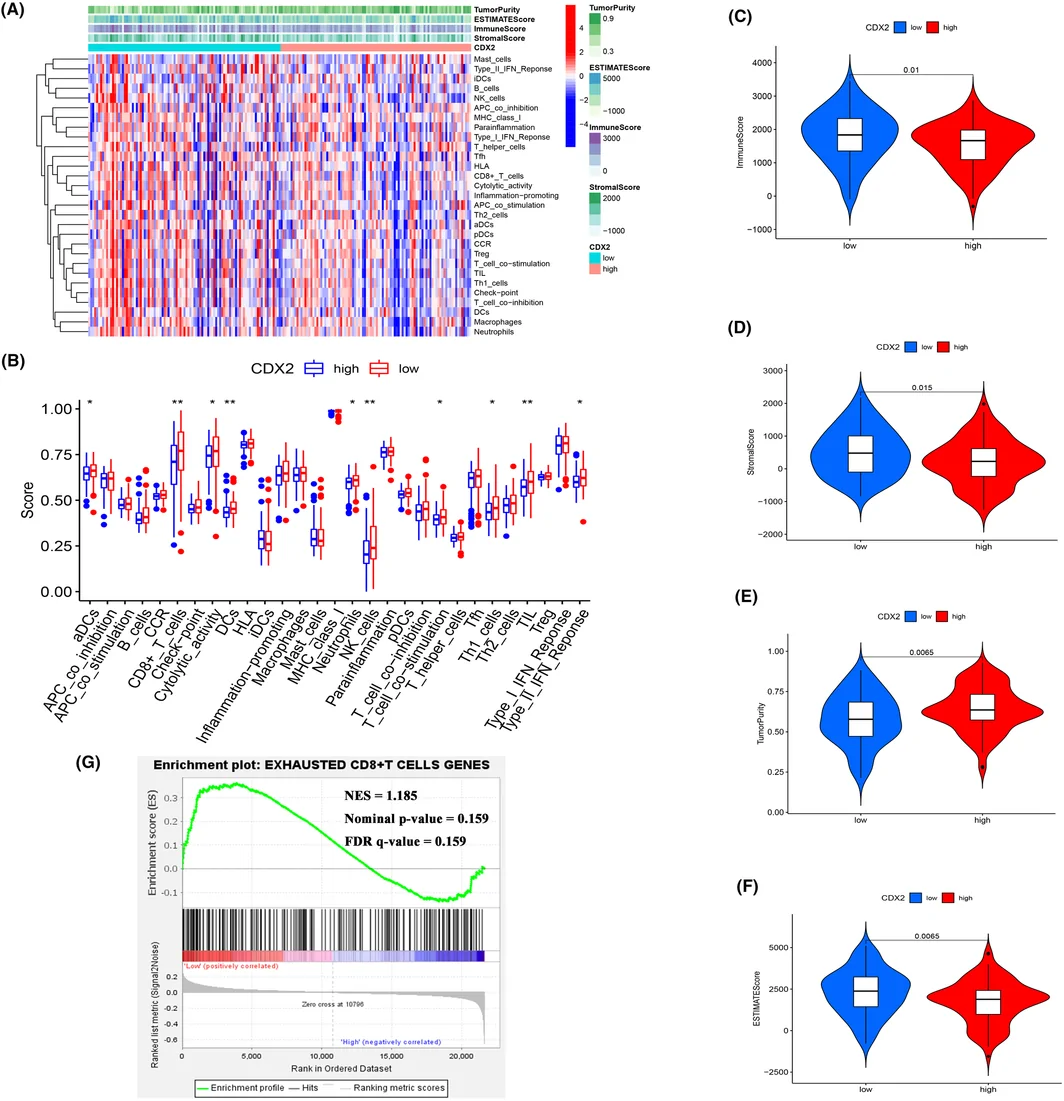
ESTIMATE algorithm identifies CDX2 expression associated with the tumor microenvironment. (A) Heatmaps displayed distinct immune cells between CDX2‐negative and CDX2‐positive subgroups. (B) The association between the infiltration levels of immune cells and the CDX2 expression level. (C–F) Comparison of the immune score (C), stromal score (D), tumor purity (E), and ESTIMATE score (F) in CDX2‐negative and CDX2‐positive patients in the ACRG cohort. (G) GSEA revealed enrichment of exhausted CD8^+^ T cell genes in tumors with low CDX2 expression. NES, normalized enrichment score. * indicates *p* < 0.05, ** indicates *p* < 0.01.

The tumor microenvironment contains stromal cells and immune cells that play a substantial role in tumor malignancy and response to chemotherapy. Therefore, it is worthwhile to further elucidate the relationship between CDX2 expression and immune microenvironment characteristics. To analyze the composition of immune cells in different CDX2 expression subgroups, we used the Wilcoxon test to compare the distribution of immune cells in different CDX2 expression subgroups. We found that CD8+ T cells, dendritic cells (DCs), natural killer (NK) cells, and tumor‐infiltrating lymphocytes (TIL) were more abundant in the CDX2‐low subgroup (all *p* < 0.01) (Figure 7A,B). To validate our findings, IHC was performed to detect the expression of CD8 in representative patients with GC. Representative immunohistochemical images are shown in Figure S5A. The IHC score was significantly higher in the CDX2‐negative group than in the CDX2‐positive group (Figure S5B). Stromal score and tumor purity represent the percentages of stromal cells and tumor cells in the tumor microenvironment, respectively. Moreover, the immune score and ESTIMATE score represent the percentage of immune cells in the tumor microenvironment and tumor purity, respectively. The CDX2‐low subgroup had relatively lower tumor purity, and higher stromal score, immune score, and ESTIMATE score (all *p* < 0.05) (Figure 7C–F). Taken together, these results suggested that the stromal cells and infiltration of immune cells in gastric adenocarcinoma increased in the CDX2‐low subgroup. We further investigated the relationship between CDX2 status and the functional status of CD8^+^ T cells. By means of GSEA, the exhausted CD8^+^ T cell gene set was found to be significantly enriched in the CDX2‐low subgroup, compared with tumors in the CDX2‐high subgroup (Figure 7G).

## 4 DISCUSSION

To identify the prognostic and predictive value of CDX2 and mucin expression in gastric adenocarcinoma, we prospectively performed CDX2 and mucin protein immunohistochemical staining in a large population sample (*n* = 782). In these analyses, the expression level of CDX2 showed a profound effect on survival and the efficacy of fluorouracil‐based postoperative chemotherapy. The main results of the present study were also validated through an independent validation cohort (*n* = 386) and an external public dataset (*n* = 193), and the findings were consistent. Similar to CDX2 expression in colorectal cancer,^4^, ^20^ we found in our present study that patients with loss of CDX2 expression tumors had poor clinical outcomes in the absence of systemic therapy, but many of them benefited greatly from postoperative adjuvant chemotherapy; despite better prognosis, patients with CDX2‐positive tumors were far less sensitive to systemic chemotherapy and patients with CDX2‐positive tumors did not benefit from adjuvant chemotherapy.

In terms of histological staining, the tissue sections were stained clearly, and no nonspecific immunoreactivity or background reactivity was observed with the monoclonal CDX2 and mucin antibodies used in our study. The results showed excellent interobserver agreement. Therefore, immunohistochemical scoring of CDX2 expression is easy to evaluate and highly reproducible, making it very suitable for clinical diagnosis. These results are supported by those of previous studies on colorectal cancer.^4^ In our study, CDX2 expression was detected in 59.7% of the evaluation cohort and 50.5% of the validation cohort. This rate is similar to that previously reported in GC using IHC (reviewed in^9^). The relationship between CDX2 expression and clinicopathological characteristics remains controversial. Our conclusion is supported by different research groups. We showed that gastric adenocarcinoma without CDX2 expression was associated with an increased likelihood of poor differentiation, which is in concordance with several previous studies.^5^, ^9^ Inconsistent with our findings, several studies showed that CDX2 expression was significantly associated with sex,^21^ vascular invasion,^22^ and tumor stage.^23^, ^24^ Meanwhile, some studies also support our findings of no association of CDX2 expression with sex,^24^ clinical stage,^25^, ^26^ or vascular invasion.^16^, ^21^, ^27^ Based on the large sample size of our study, we replicated our findings in different cohorts. Therefore, these results support the reliability of our findings.

Although previous reports have examined the correlation between CDX2 and mucin expression, the majority of the studies were characterized by small sample sizes.^16^, ^26^ The sample size of our study was the largest reported to date in this field (1361 cases). Furthermore, serial sections were excised from the same tumor blocks for prospective CDX2 and mucin analyses in this study. This method can effectively avoid interference from intratumoral heterogeneity. Of the four mucin markers we examined, CDX2 expression was strongly associated with intestinal‐type mucin (MUC2 and CD10) expression in tumors. These results are similar to those of previous studies reporting that 70%–80% of tumors with CD10 and MUC2 expression showed CDX2 expression.^16^, ^28^ Consistent with some previous reports,^16^, ^29^ we also found that CDX2 expression was significantly increased in the intestinal and gastrointestinal phenotypes compared with the other two phenotypes. Survival analysis showed that only high MUC5AC protein expression was related to a good prognosis. This result is consistent with some previous studies reporting that GC patients with high expression of MUC5AC have a good prognosis compared with those with high expression of intestinal mucin makers.^19^, ^30^ The mechanism of poorer outcomes in patients with an intestinal mucin phenotype is complicated and may be partially explained by a higher rate of genetic alteration, which has been associated with more aggressive disease. For example, *TP53* mutation and chromosomal alteration are detected more frequently in the intestinal phenotype of GC.^10^, ^30^ In addition, GC with an intestinal mucin phenotype has been shown to display more nuclear β‐catenin expression compared, also suggesting a more aggressive behavior.^31^ Subsequent planned subgroup analyses showed no significant interaction between treatment effect on prognosis and mucin protein expression. While an early study reported that mucin expression was predictive of the efficacy of 5‐fluorouracil‐based postoperative chemotherapy (*n* = 137),^32^ it had a small sample size, and interaction analysis was not performed, which might be the potential reason for the inconsistent results.

In our study, we showed that loss of CDX2 expression was not significantly associated with poor outcomes in the whole cohort. However, in the surgery‐only subgroup, loss of CDX2 expression allowed us to identify a group of patients with a particularly poor prognosis. To the best of our knowledge, this is the first study to confirm the clinical significance of CDX2 expression in chemoresistance in gastric cancer. Recently, Dalerba and colleagues demonstrated that without adjuvant chemotherapy, CDX2‐negative tumors were associated with a low 5‐year DFS rate among colon cancer patients.^4^ A cohort of patients who have not received adjuvant chemotherapy is ideal for testing prognostic markers because they represent the natural history of cancer. As shown in our study, there is a cross‐effect between adjuvant chemotherapy and the prognostic value of CDX2, and loss of CDX2 is associated with benefits from adjuvant chemotherapy.^4^ The correlation between CDX2 status and survival may be confounded by adjuvant chemotherapy. This may partially explain why the prognostic value of CDX2 status in patients with GC remains controversial. This pattern has also been observed with other biomarkers in patients with GC.^33^, ^34^ CDX2 is a key regulator of intestinal development and homeostasis and is also involved in intestinal‐type tumors, such as GC. Intestinal metaplasia is a well‐established precursor for intestinal‐type GC, and it is, at least in part, caused by *H. pylori*‐induced expression of CDX2.^35^ Our results showed that patients with *H. pylori* infection had a significantly higher prevalence of positive CDX2 expression (~70% positivity) than those without *H. pylori* infection (~20% positivity) (data not shown). However, loss of CDX2 enhances the invasive potential of gastric tumor cells. This is supported by publications describing that overexpression of CDX2 in tumor cells deficient in CDX2 exerts tumor suppressor activity by inducing intestinal differentiation,^36^ reducing cell proliferation,^37^ and reversing epithelial‐to‐mesenchymal transition.^38^

The current work demonstrated that the benefit of adjuvant chemotherapy observed in CDX2‐negative patients was superior to that observed in CDX2‐positive patients. To our knowledge, no prior study has specifically reported an association between CDX2 expression and benefits from adjuvant chemotherapy in GC patients. Similarly, Fernandez et al.^39^ showed a significant association between CDX2‐positive expression and poor regression in gastric carcinoma patients who received neoadjuvant therapy. Moreover, consistent with our findings in clinical patients, loss of CDX2 expression was strongly associated with sensitivity to chemotherapeutics in vitro,^40^ and overexpression of CDX2 promoted the development of multidrug resistance.^41^ Previous studies addressing the role of CDX2 in inducing multidrug resistance have clearly shown that this transcription factor is likely important for the basal expression of genes that control susceptibility.^40^, ^42^, ^43^, ^44^, ^45^ The multidrug resistance genes Reg IV,^42^, ^44^, ^45^ CFTR and P‐gp^40^, ^43^ can be positively regulated by CDX2. In this study, positive correlations in the expression levels of mRNA were observed between CDX2 and its target multidrug resistance genes. It is possible that CDX2 induces the expression of multidrug resistance genes, resulting in relatively no benefits from adjuvant chemotherapy.

In recent years, individualized chemotherapy based on molecular typing has had an important role in the treatment of specific cancers. Based on an integrative analysis of genomic alterations, the TCGA team proposed four molecular subtypes of GC: EBV positive, microsatellite unstable (MSI), genomically stable, and chromosomal instability.^46^ In this genomic typing, two notable subtypes are the EBV and MSI subtypes. The EBV subtype is associated with high expression of the immune checkpoints, PD1, PDL1, and PDL2. The MSI subtype is associated with a high tumor mutational burden. These two subtypes have a better response to immunotherapy. However, few studies have shown that TCGA typing can be used to predict sensitivity to conventional chemotherapy. In addition, the technological complexity of TCGA typing has limited its clinical application. In the present study, our findings may provide an easy‐to‐use marker for individual adjuvant chemotherapy in patients with GC. It still needs to be verified by prospective clinical studies.

The tumor microenvironment heavily influences patient survival and the response to tumor therapy. In 2013, the ESTIMATE algorithm was first established by Yoshihara et al. to evaluate tumor purity, infiltrating immune cells, and the presence of stromal cells.^47^ This algorithm will help assess the composition of the tumor microenvironment. It generates three scores: Stromal score (the presence of stromal cells), Immune score (the presence of immune cells), and ESTIMATE score (the tumor purity).^47^ In our study, the CDX2‐low subgroup had relatively lower tumor purity, higher stromal score, and ESTIMATE score. Multiple studies have now reported that low tumor purity is associated with a malignant phenotype and poor prognosis.^48^, ^49^, ^50^ This could partially explain why patients with low CDX2 expression had a significantly poor prognosis. However, higher immune cell infiltration and activation were also found in the low CDX2 expression subgroup. Not only a higher immune score but also CD8+ T cells, DCs, NK cells, and TIL were more enriched in the CDX2 low subgroup. A substantial body of research has revealed that primary tumors with a robust immune infiltrate are associated with a favorable prognosis and better response to chemotherapy.^51^, ^52^, ^53^ Therefore, we suggest that the better response to chemotherapy in the low CDX2 expression subgroup might result from a better immune microenvironment.

Several limitations of this study should be noted. First, the CDX2 IHC data analyzed in our study were obtained from only a single medical center. To try to offset this limitation, we included an independent ACRG cohort to reconfirm our findings. Second, because the time to initiation of adjuvant chemotherapy was not available for all patients in this study, we did not exclude the patients with a very long interval between surgery and initiation of adjuvant therapy. Analysis of the available data showed that above 85% of the patients who received adjuvant chemotherapy had initiated treatment within 8 weeks after radical gastrectomy. The proportion of patients with a very long interval between surgery and initiation of adjuvant therapy is low. Third, since this is an observational study, there were no restrictions on chemotherapy regimens. The ACRG cohort was similar and included patients who received different chemotherapy regimens. We failed to analyze the effect of chemotherapy cycles on the results. In addition, because of the observational design of the study, residual confounding is possible, unlike in randomized studies. Given the single center and observational nature of our study, these findings will need to be further validated to elucidate the clinical value of CDX2 in daily clinical practice.

## 5 CONCLUSION

Our study clarified that loss of CDX2 expression is quite a frequent event in gastric adenocarcinoma. Although the loss of CDX2 is an independent risk factor for survival in stage II and III GC patients who do not receive adjuvant chemotherapy, it is associated with benefits from adjuvant chemotherapy. These findings provide a novel simple biomarker for individual adjuvant chemotherapy in patients with GC. Routine analysis of CDX2 staining is useful for selecting the most suitable patients for adjuvant chemotherapy to prevent recurrence. Our findings warrant further validation through future randomized adjuvant trials.

## AUTHOR CONTRIBUTIONS

**Xianchun Gao:** Conceptualization (equal); data curation (equal); formal analysis (equal); funding acquisition (supporting); writing – original draft (equal). **Weili Han:** Data curation (equal); formal analysis (equal); writing – original draft (equal). **Ling Chen:** Formal analysis (equal); methodology (equal); software (equal). **Hongwei Li:** Methodology (lead); resources (lead). **Fenli Zhou:** Methodology (lead). **Bin Bai:** Data curation (supporting); methodology (supporting). **Junya Yan:** Data curation (supporting). **Yong Guo:** Data curation (supporting); methodology (supporting). **Kun Liu:** Methodology (supporting). **Wenjiao Li:** Data curation (supporting). **Renlong Li:** Data curation (supporting). **Qiangqiang Yuan:** Data curation (supporting). **Jiehao Zhang:** Data curation (supporting); formal analysis (supporting). **Yuanyuan Lu:** Data curation (supporting). **Xiaodi Zhao:** Data curation (supporting). **Gang Ji:** Data curation (supporting). **Mengbin Li:** Data curation (supporting). **Qingchuan Zhao:** Data curation (supporting); supervision (supporting). **Kaichun Wu:** Conceptualization (lead); supervision (supporting). **Zengshan Li:** Methodology (lead); supervision (equal); writing – review and editing (lead). **Yongzhan Nie:** Conceptualization (equal); formal analysis (equal); funding acquisition (equal); project administration (equal); supervision (equal); writing – review and editing (equal).

## CONFLICT OF INTEREST STATEMENT

The authors declare no conflicts of interest.

## DISCLOSURE

The authors declare that they have no known competing financial interests or personal relationships that could have appeared to influence the work reported in this paper.

## ETHICS STATEMENT

All human tissue experiments were approved by the Ethical Committee of Xijing Hospital. All procedures were in accordance with the ethical standards of the responsible committee on human experimentation (institutional and national) and with the Helsinki Declaration of 1964 and later versions. Informed consent to be included in the study, or the equivalent, was obtained from all patients.

## REGISTRY AND THE REGISTRATION NO. OF THE STUDY

Not Applicable.

## ANIMAL STUDIES

Not Applicable.

## Supporting information


Figure S1.

Figure S2.

Figure S3.

Figure S4.

Figure S5.

Table S1–S4.

## Data Availability

The datasets used and/or analyzed during the current study are available from the corresponding author on reasonable request.
