# Supplementary figures and images for: Association of CDX2 and mucin expression with chemotherapeutic benefits in patients with stage II/III gastric cancer
Source: Cancer Med. 2023 Aug 21;12(17):17613–31. doi: 10.1002/cam4.6379 (PMC10523976; doi:10.1002/cam4.6379)

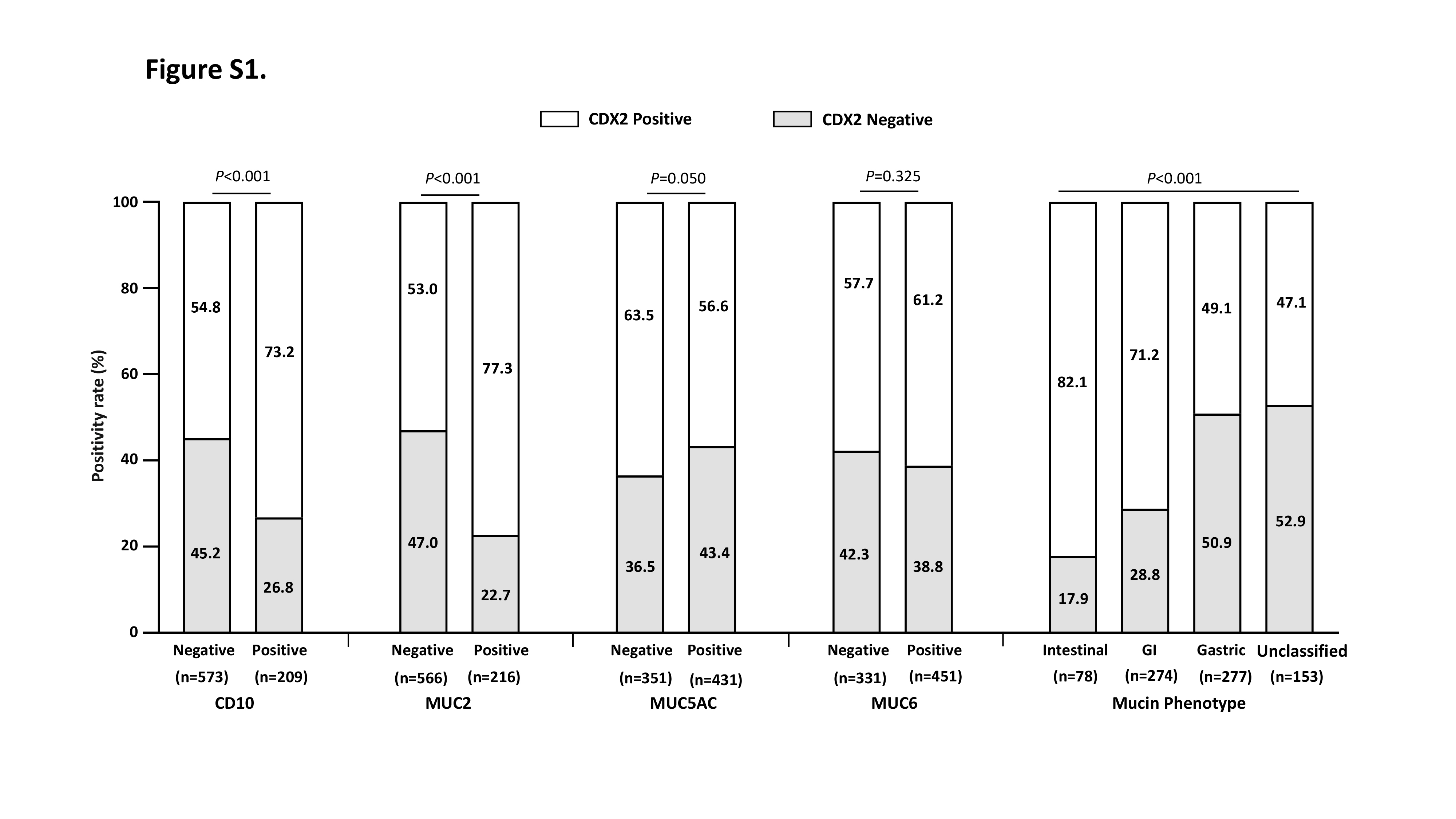

Supplement: Supplementary file 1 — Figure S1. [file CAM4-12-17613-s001.jpg]

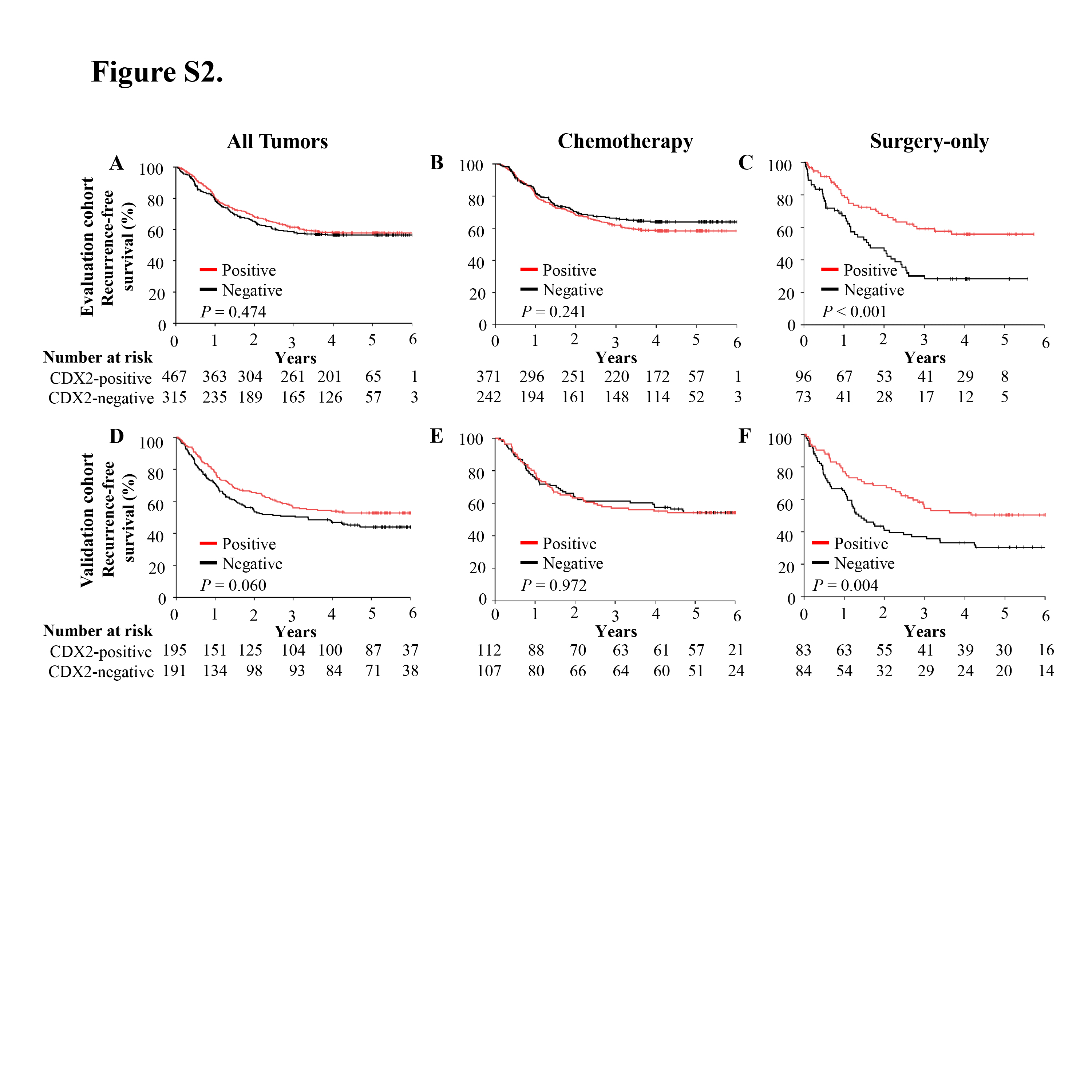

Supplement: Supplementary file 2 — Figure S2. [file CAM4-12-17613-s006.jpg]

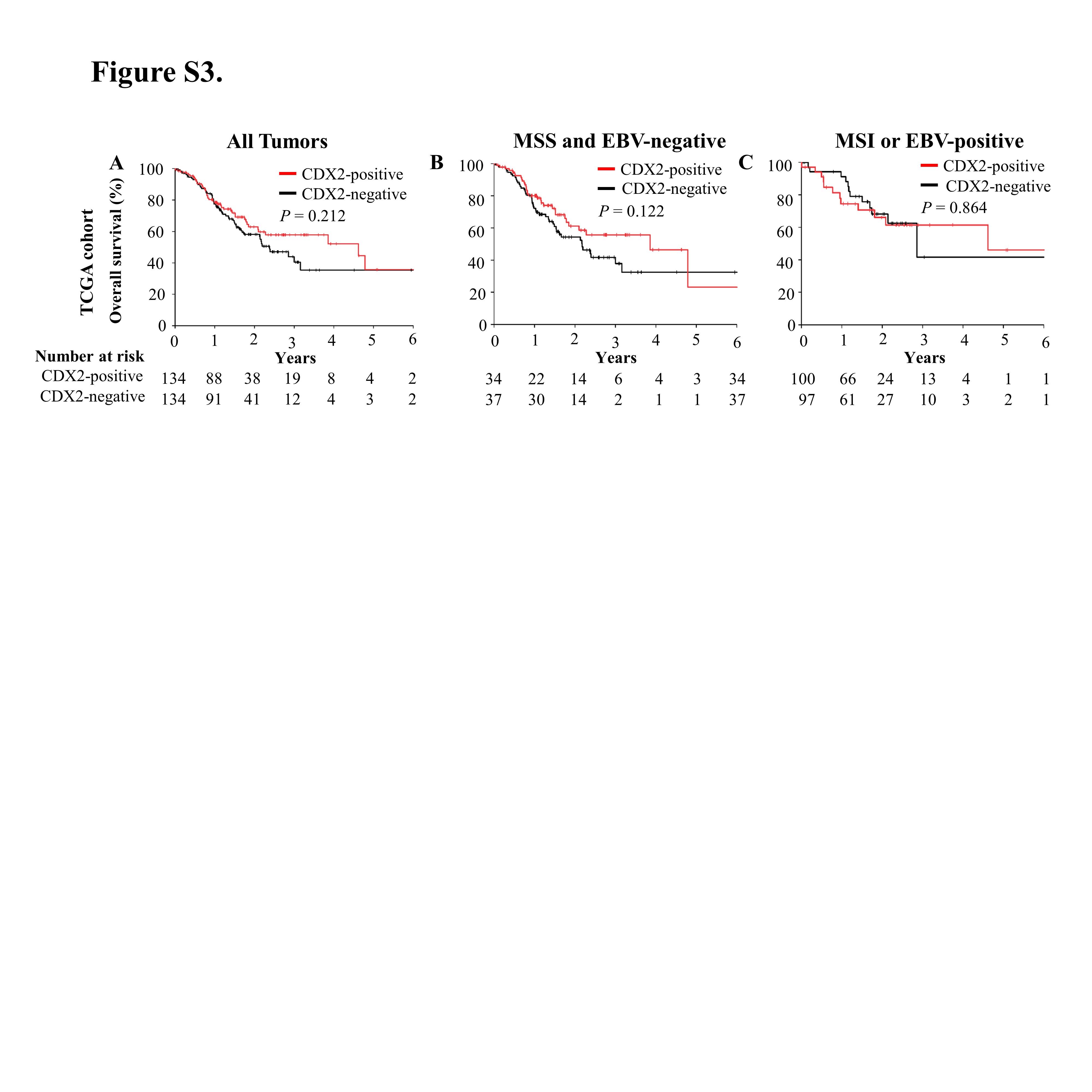

Supplement: Supplementary file 3 — Figure S3. [file CAM4-12-17613-s003.jpg]

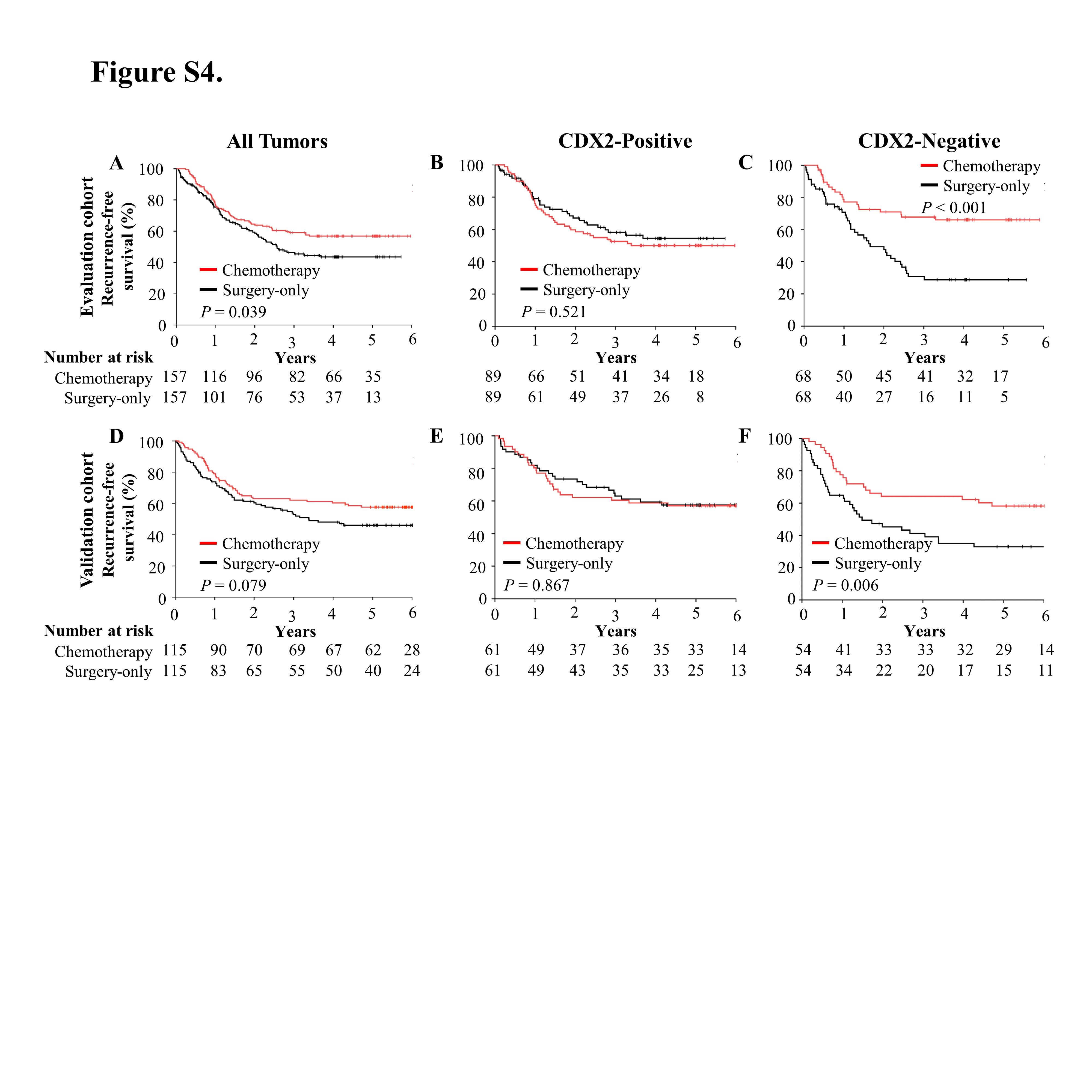

Supplement: Supplementary file 4 — Figure S4. [file CAM4-12-17613-s002.jpg]

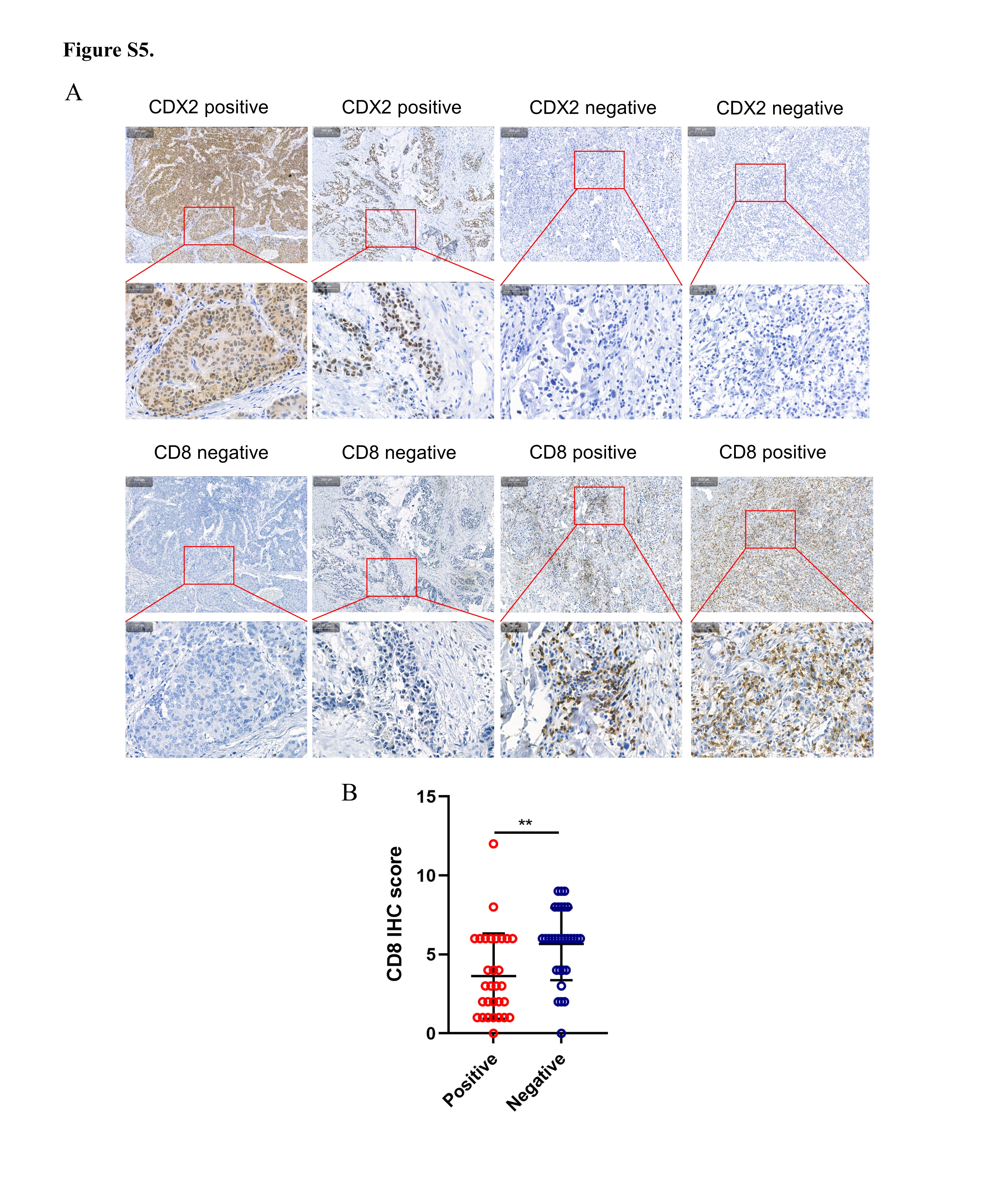

Supplement: Supplementary file 5 — Figure S5. [file CAM4-12-17613-s005.jpg]
