# Supplementary material for: Association of CDX2 and mucin expression with chemotherapeutic benefits in patients with stage II/III gastric cancer
Source: Cancer Med. 2023 Aug 21;12(17):17613–31. doi: 10.1002/cam4.6379 (PMC10523976; doi:10.1002/cam4.6379)
Supplement: Supplementary file 6 — Table S1–S4. [file CAM4-12-17613-s004.docx]

**Supplemental Information**

**Table S1. Interobserver agreement in immunohistochemistry results of CDX2 in the validation cohort (n = 386).**

| CDX2 score  of individual tissue cores | | Observer #1 | | |
| --- | --- | --- | --- | --- |
|  |  | Score 0 | Score 1 | Score 2 |
| Observer #2 | Score 0 | 120 | 5 | 0 |
|  | Score 1 | 8 | 52 | 8 |
|  | Score 2 | 0 | 6 | 187 |

**Table S2. Clinical and demographic variables according to the chemotherapy in evaluation cohort.**

|  | **Before propensity score matching** | | |  | **After propensity score matching** | | |
| --- | --- | --- | --- | --- | --- | --- | --- |
| **Parameter** | **Surgery-only**  **n=169 (%)** | **Chemotherapy**  **n=613 (%)** | ***P* value** |  | **Surgery-only**  **n=157 (%)** | **Chemotherapy**  **n=157 (%)** | ***P* value** |
| Age (years) |  |  | <0.001 |  |  |  | 0.572 |
| ≥65 | 82 (48.5) | 131 (21.4) |  |  | 71 (45.2) | 76 (48.4) |  |
| <65 | 87 (51.5) | 482 (78.6) |  |  | 86 (54.8) | 81 (51.6) |  |
| Sex |  |  | 0.686 |  |  |  | 0.096 |
| Male | 131 (77.5) | 466 (76.0) |  |  | 122 (77.7) | 109 (69.4) |  |
| Female | 38 (22.5) | 147 (24.0) |  |  | 35 (22.3) | 48 (30.6) |  |
| Primary tumor location |  |  | 0.322 |  |  |  | 0.476 |
| Gastric | 107 (63.3) | 413 (67.4) |  |  | 100 (63.7) | 106 (67.5) |  |
| GE junction | 62 (36.7) | 200 (32.6) |  |  | 57 (36.3) | 51 (32.5) |  |
| Histopathological differentiation |  |  | 0.520 |  |  |  | 0.656 |
| High/middle | 27 (16.0) | 111 (18.1) |  |  | 26 (16.6) | 29 (18.5) |  |
| Poor or Mucinous | 142 (84.0) | 502 (81.9) |  |  | 131 (83.4) | 128 (81.5) |  |
| Perineural invasion |  |  | 0.222 |  |  |  | 0.427 |
| No | 7 (4.1) | 41 (6.7) |  |  | 6 (3.8) | 9 (5.7) |  |
| Yes | 162 (95.9) | 572 (93.3) |  |  | 151 (96.2) | 148 (94.3) |  |
| Lymphovascular invasion |  |  | 0.094 |  |  |  | 0.395 |
| No | 29 (17.2) | 142 (23.2) |  |  | 28 (17.8) | 34 (21.7) |  |
| Yes | 140 (82.8) | 471 (76.8) |  |  | 129 (82.2) | 123 (78.3) |  |
| Examined lymph nodes |  |  | 0.045 |  |  |  | 0.779 |
| ≥ 20 | 127 (75.1) | 503 (82.1) |  |  | 124 (79) | 126 (80.3) |  |
| < 20 | 42 (24.9) | 110 (17.9) |  |  | 33 (21) | 31 (19.7) |  |
| Pathologic stage (AJCC 8th) |  |  | 0.114 |  |  |  | 0.534 |
| Stage II | 47 (27.8) | 210 (34.3) |  |  | 43 (27.4) | 48 (30.6) |  |
| Stage III | 122 (72.2) | 403 (65.7) |  |  | 114 (72.6) | 109 (69.4) |  |

Abbreviation: GE junction, gastroesophageal junction.

**Table S3. Clinical and demographic variables according to the chemotherapy in validation cohort.**

|  | **Before propensity score matching** | | |  | **After propensity score matching** | | |
| --- | --- | --- | --- | --- | --- | --- | --- |
| **Parameter** | **Surgery-only**  **n=167 (%)** | **Chemotherapy**  **n=219 (%)** | ***P* value** |  | **Surgery-only**  **n=115 (%)** | **Chemotherapy**  **n=115 (%)** | ***P* value** |
| Age (years) |  |  | <0.001 |  |  |  | 0.498 |
| ≥65 | 78 (46.7) | 58 (26.5) |  |  | 42 (36.5) | 47 (40.9) |  |
| <65 | 89 (53.3) | 161 (73.5) |  |  | 73 (63.5) | 68 (59.1) |  |
| Sex |  |  | 0.541 |  |  |  | 0.427 |
| Male | 131 (78.4) | 166 (75.8) |  |  | 92 (80.0) | 87 (75.7) |  |
| Female | 36 (21.6) | 53 (24.2) |  |  | 23 (20.0) | 28 (24.3) |  |
| Primary tumor location |  |  | 0.731 |  |  |  | 0.475 |
| Gastric | 107 (64.1) | 144 (65.8) |  |  | 77 (67.0) | 82 (71.3) |  |
| GE junction | 60 (35.9) | 75 (34.2) |  |  | 38 (33.0) | 33 (28.7) |  |
| Histopathological differentiation |  |  | 0.429 |  |  |  | 0.397 |
| High/middle | 59 (35.3) | 69 (31.5) |  |  | 40 (34.8) | 34 (29.6) |  |
| Poor or Mucinous | 108 (64.7) | 150 (68.5) |  |  | 75 (65.2) | 81 (70.4) |  |
| Perineural invasion |  |  | 0.006 |  |  |  | 1.000 |
| No | 28 (16.8) | 17 (7.8) |  |  | 13 (11.3) | 13 (11.3) |  |
| Yes | 139 (83.2) | 202 (92.2) |  |  | 102 (88.7) | 102 (88.7) |  |
| Lymphovascular invasion |  |  | 0.066 |  |  |  | 0.781 |
| No | 62 (37.1) | 62 (28.3) |  |  | 38 (33.0) | 40 (34.8) |  |
| Yes | 105 (62.9) | 157 (71.7) |  |  | 77 (67.0) | 75 (65.2) |  |
| Examined lymph nodes |  |  | <0.001 |  |  |  | 0.654 |
| ≥ 20 | 137 (82) | 205 (93.6) |  |  | 103 (89.6) | 105 (91.3) |  |
| < 20 | 30 (18) | 14 (6.4) |  |  | 12 (10.4) | 10 (8.7) |  |
| Pathologic stage (AJCC 8th) |  |  | 0.792 |  |  |  | 0.766 |
| Stage II | 46 (27.5) | 63 (28.8) |  |  | 30 (26.1) | 32 (27.8) |  |
| Stage III | 121 (72.5) | 156 (71.2) |  |  | 85 (73.9) | 83 (72.2) |  |

Abbreviation: GE junction, gastroesophageal junction.

**Table S4. Clinical and demographic variables according to the chemotherapy in ACRG cohort.**

|  | **Before propensity score matching** | | |  | **After propensity score matching** | | |
| --- | --- | --- | --- | --- | --- | --- | --- |
| **Parameter** | **Surgery-only**  **n=88 (%)** | **Chemotherapy**  **n=105 (%)** | ***P* value** |  | **Surgery-only**  **n=70 (%)** | **Chemotherapy**  **n=70 (%)** | ***P* value** |
| Age (years) |  |  | 0.003 |  |  |  | 1.000 |
| ≥65 | 51 (58) | 38 (36.2) |  |  | 34 (48.6) | 34 (48.6) |  |
| <65 | 37 (42) | 67 (63.8) |  |  | 36 (51.4) | 36 (51.4) |  |
| Sex |  |  | 0.538 |  |  |  | 0.848 |
| Male | 61 (69.3) | 77 (73.3) |  |  | 52 (74.3) | 51 (72.9) |  |
| Female | 27 (30.7) | 28 (26.7) |  |  | 18 (25.7) | 19 (27.1) |  |
| Primary tumor location |  |  | 0.622 |  |  |  | 0.546 |
| Gastric | 79 (89.8) | 91 (87.5) |  |  | 63 (90) | 65 (92.9) |  |
| GE junction | 9 (10.2) | 13 (12.5) |  |  | 7 (10) | 5 (7.1) |  |
| Lauren’s classification |  |  | 0.917 |  |  |  | 0.756 |
| Intestinal | 45 (51.1) | 54 (51.4) |  |  | 37 (52.9) | 38 (54.3) |  |
| Diffuse | 40 (45.5) | 48 (45.7) |  |  | 31 (44.3) | 31 (44.3) |  |
| Mixed | 3 (3.4) | 3 (2.9) |  |  | 2 (2.9) | 1 (1.4) |  |
| Pathologic stage (AJCC 6th) |  |  | 0.823 |  |  |  | 0.865 |
| Stage II | 45 (51.1) | 52 (49.5) |  |  | 38 (54.3) | 37 (52.9) |  |
| Stage III | 43 (48.9) | 53 (50.5) |  |  | 32 (45.7) | 33 (47.1) |  |

Abbreviation: GE junction, gastroesophageal junction.

**Figure S1** Comparison of CDX2 status and gastric/intestinal mucin markers. GI, Gastrointestinal.

**Figure S2** Relationship between CDX2 status and patient recurrence-free survival. (A-C) Kaplan-Meier plots for recurrence-free survival in the evaluation cohort (n=782) according to CDX2 status (A: all tumors, B: adjuvant chemotherapy subgroup, C: surgery-only subgroup). (D-F) Kaplan-Meier plots for recurrence-free survival in the validation cohort (n=386) according to CDX2 status (D: all tumors, E: adjuvant chemotherapy subgroup, F: surgery-only subgroup).

**Figure S3** Relationship between CDX2 status and patient overall survival based on gastric cancer data set from the TCGA database. (A) Among all patients with stage II/III gastric cancer; (B) Among patients with MSS, EBV-negative gastric cancer; (C) Among patients with MSI or EBV-positive gastric cancer.

**Figure S4** Association between CDX2 status and benefit from adjuvant chemotherapy after propensity score matching. (A-C) Kaplan-Meier plots for recurrence-free survival according to treatment in the evaluation cohort (A: all tumors, B: CDX2 positive subgroup, C: CDX2 negative subgroup). (D-F) Kaplan-Meier plots for recurrence-free survival according to treatment in the validation cohort (D: all tumors, E: CDX2 positive subgroup, F: CDX2 negative subgroup).

**Figure S5.** CD8 is related to the decreased CDX2 expression. (A) Representative immunohistochemical images for CDX2 and CD8 IHC in serial tumor sections. (B) The IHC score was significantly higher in the CDX2-negative group than in the CDX2-positive group. The IHC scores are the average of the score of T cell staining multiplied by the score of staining intensity. According to the intensity of CD8+ T cell staining from loss to strong, it is scored from 0 to 3 points; according to the proportion of positive cells from 0 to 100% it is scored from 0 to 4 points. ***P* < 0.01.
